# Blocking ACSL6 Compromises Autophagy via FLI1‐Mediated Downregulation of COLs to Radiosensitize Lung Cancer

**DOI:** 10.1002/advs.202403202

**Published:** 2024-08-29

**Authors:** Wen Ding, Shijun Bao, Qingwei Zhao, Wei Hao, Kai Fang, Yanlan Xiao, Xiaoting Lin, Zhemeng Zhao, Xinyi Xu, Xinyue Cui, Xiwen Yang, Liuhuan Yao, Hai Jin, Kun Zhang, Jiaming Guo

**Affiliations:** ^1^ Department of Radiation Medicine College of Naval Medicine Naval Medical University Shanghai 200433 P. R. China; ^2^ Department of Medicine College Jiangnan University Wuxi Jiangsu 214000 P. R. China; ^3^ College of Basic Medicine Naval Medical University Shanghai 200433 P. R. China; ^4^ Department of Cardiothoracic Surgery Changhai Hospital Naval Medical University Shanghai 200433 P. R. China; ^5^ Department of Laboratory Medicine and Central Laboratory Sichuan Academy of Medical Sciences Sichuan Provincial People's Hospital School of Medicine University of Electronic Science and Technology of China No. 32, West Second Section, First Ring Road Chengdu Sichuan 610072 P. R. China

**Keywords:** autophagy, collagen, FLI1, long‐chain Acyl‐CoA Synthase 6, lung cancer, radiosensitivity, radiotherapy

## Abstract

Lung cancer (LC) is the leading cause of cancer‐related mortality worldwide. Radiotherapy is the main component of LC treatment; however, its efficacy is often limited by radioresistance development, resulting in unsatisfactory clinical outcomes. Here, we found that LC radiosensitivity is up‐regulated by decreased expression of long‐chain acyl‐CoA synthase 6 (ACSL6) after irradiation. Deletion of ACSL6 results in significant elevation of Friend leukemia integration 1 transcription factor (FLI1) and a marked decline of collagens (COLs). Blocking of ACSL6 impairs the tumor growth and upregulates FLI1, which reduces the levels of COLs and compromises irradiation‐induced autophagy, leading to considerable therapeutic benefits during radiotherapy. Moreover, the direct interaction between ACSL6 and FLI1 and engagement between FLI1 and COLs indicates the involvement of the ACSL6‐FLI1‐COL axis. Finally, the potently adjusted autophagy flux reduces its otherwise contributive capability in surviving irradiation stress and leads to satisfactory radiosensitization for LC radiotherapy. These results demonstrate that enhanced ACSL6 expression promotes the aggressive performance of irradiated LC through increased FLI1‐COL‐mediated autophagy flux. Thus, the ACSL6‐FLI1‐Col‐autophagy axis may be targeted to enhance the radiosensitivity of LC and improve the management of LC in radiotherapy.

## Graphical abstract

Polishing lung cancer (LC) radiosensitivity benefits its radiotherapy. Irradiation is shown to up‐regulates long‐chain acyl‐CoA synthase 6 (ACSL6), strengthening its binding to Friend leukemia integration 1 transcription factor (FLI1), hindering FLI1's nuclear translocation and releasing its transcriptional inhibition of key collagens. Consequently, the autophagy is boosted, causing the radioresistance development. ACSL6 can be a new target for bettering LC radiotherapy.

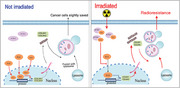

## 1 Introduction

Lung cancer (LC) is the leading cause of cancer‐related deaths worldwide, with poor 5‐year survival rates of 4–17%.^[^ ^1^, ^2^ ^]^ Approximately 85% of patients with LC are diagnosed with non‐small cell lung cancer, comprising lung adenocarcinoma (LUAD) and lung squamous cell carcinoma (LUSC).^[^ ^3^ ^]^ Among the major therapeutic modalities, radiotherapy is crucial in LC treatment, with ≈77% of patients requiring it through the course of treatment.^[^ ^4^ ^]^ However, the inevitable occurrence of radioresistance (RR) severely compromises the curative effect of radiotherapy, accounting for a very low 5‐year survival rate of 10–15%.^[^ ^5^, ^6^, ^7^ ^]^ Hence, improving lung cancer radiosensitivity (LCRS) is vital for radiotherapeutic benefits, and has attracted the attention of researchers for a long time.

The contributors to the development of RR include inherent and acquired mechanisms, the latter of which play major roles in this process, such as DNA damage repair, tumor microenvironment (TME) remodeling, and autophagy.^[^ ^8^, ^9^, ^10^, ^11^, ^12^ ^]^ Considerable advances have been made to overcome RR based on these mechanisms.^[^ ^13^ ^]^ For instance, we showed that polydatin radiosensitizes LC by modulating tumor‐infiltrating B cells.^[^ ^14^ ^]^ LC radiosensitizers of different origins have also been developed by other groups.^[^ ^15^, ^16^ ^]^ However, considerable gaps in the mechanistic understanding of RR need to be filled to develop effective radiosensitizers.

Metabolic reprogramming is a hallmark of tumor progression in solid tumors, including LC, and is mainly characterized by aerobic glycolysis.^[^ ^17^ ^]^ Recent reports indicate that lipid metabolism, particularly fatty acid (FA) metabolism, is frequently altered in LC, contributing significantly to malignant phenotypes such as RR.^[^ ^18^, ^19^, ^20^, ^21^ ^]^ FAs are converted to fatty acyl‐CoA esters by acyl‐CoA synthases (ACSs) before they are channeled toward anabolic or catabolic pathways. ACSs include short‐chain ACSs (ACSS), medium‐chain ACSs (ACSM), long‐chain ACSs (ACSL), and very long‐chain ACSs (ACSVL), named after the carbon chain length of their respective substrate,^[^ ^22^ ^]^ among which the ACSL family stands out for its involvement in multiple metabolic fates and their various intricate relationships with cancer.^[^ ^23^, ^24^ ^]^ Compared with other isoenzymes (ACSL1 and ACSL3–5), the role of ACSL6 in cancer has not been fully investigated.^[^ ^24^ ^]^

Autophagy is an evolutionarily conserved mechanism that maintains cellular homeostasis by eradicating injured organelles and recycling them, thereby, helping them survive crises triggered by various stimuli including ionizing radiation (IR).^[^ ^25^ ^]^ As a delicate cascade, autophagy consists of several steps, including induction, nucleation, elongation, and maturation. Autophagosome formation relies on ubiquitin‐like conjugation systems, involving autophagy related 12 (ATG12) and microtubule associated protein 1 light chain 3 (LC3).^[^ ^26^ ^]^ Autophagy consumes large amounts of energy and lipid building blocks. Accumulating evidence indicates that alterations in FA metabolism, such as changes in the expression of ACSLs, markedly affect autophagic flux within cancer cells, resulting in the modulation of the malignant phenotype.^[^ ^27^, ^28^ ^]^ IR‐induced autophagy often renders cancer cells more resistant to radiotherapy. Thus, many researchers have explored strategies to radiosensitize LC by inhibiting IR‐induced autophagy.^[^ ^29^, ^30^ ^]^ Therefore, whether there is deeper and more sophisticated communication among IR‐induced autophagy, lipid remodeling, and acquired RR remains to be determined.

Recently, we discovered a distinct radiobiological response of ACSL6 and found that only this specific isoenzyme was significantly upregulated at 24 and 48 h after IR. In the present study, we investigated the role of ASCL6 in RR using comprehensive methods such as establishing a genetically engineered mouse model (GEMM). We show a hitherto unknown RR mechanism namely the Friend leukemia integration 1 transcription factor (FLI1)‐ollagen (COL)‐autophagy cascade, which is initiated by the IR‐provoked ACSL6 leading to its inhibition of FLI1, which then relieves its transcriptional suppression of COLs whereby the IR‐evoked autophagy rises with it and the LCRS compromised. Our findings have potential therapeutic implications for sensitizing LC for radiotherapy in patients.

## 2 Results

### 2.1 Decreased ACSL6 Expression Increases the Radiosensitivity of Lung Carcinoma

**Figure 1:**
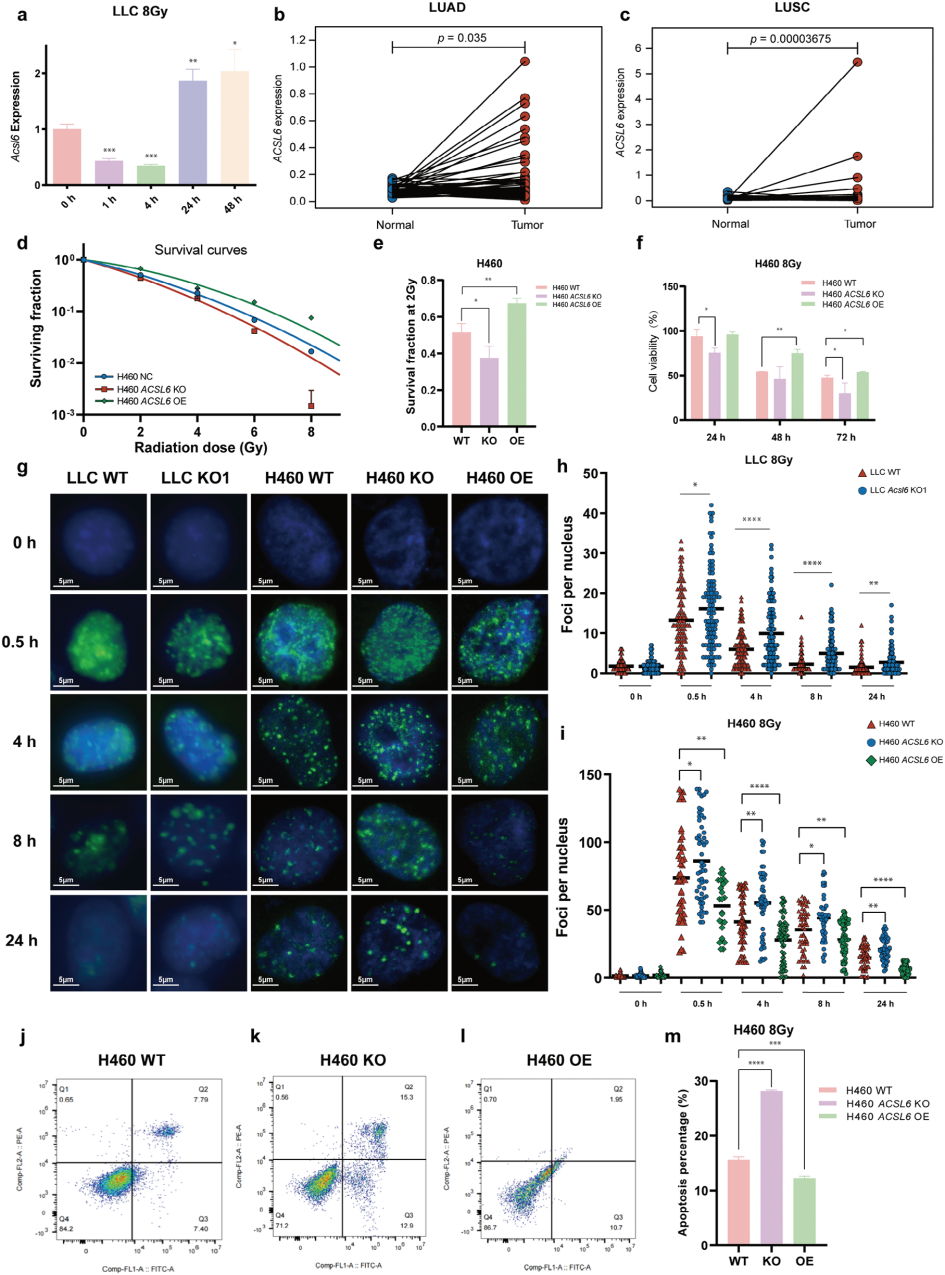
ACSL6, which is upregulated upon ionizing radiation (IR) treatment and is highly expressed in lung tumor tissue, plays a vital role in sustaining radioresistance of lung cancer. a) Expression of *ACSL6* in LLC at the indicated time points post IR treatment, as determined using RT‐qPCR (*n* = 4 per group). b,c) The in‐pair analysis of differences in *ACSL6* expression between tumor and normal tissues in the same patient in the Cancer Genome Atlas database. LUAD, lung adenocarcinoma; LUSC, lung squamous cell carcinoma. d) Loss of *ACSL6* decreased the surviving fraction of H460 cells, as determined using the clonogenic assay after delivery of different IR doses, whereas overexpression of *ACSL6* increased it. Note that the surviving fraction was measured using the linear quadratic equation model (*n* = 3 for each replicate). e) Bar graph showing the surviving fraction at 2 Gy of H460 cells (*n* = 3 per group). f) Viability of H460 cells, determined using the CCK8 assay (*n* = 6 per group). g) Representative images of immunofluorescence staining of γ‐H2AX foci in LLC and H460 cells at 8 Gy. h,i) Bar graphs showing the mean number of foci per nucleus for LLC and H460 cells at the indicated times points after IR treatment (at least 50 cells per group were included). j–m) Apoptosis of H460 cells at 24 h after IR treatment, as determined using flow cytometry after Annexin V FITC/PI double staining. Bar graph shows the apoptosis percentage (*n* = 3 per group). Data are presented as mean ± SEM. The two‐tailed independent Student's *t*‐test was used for comparison between two groups. *p < 0.05, **p < 0.01, ***p < 0.001, ****p < 0.0001.

As alterations in cellular metabolism, including aerobic glycolysis,^[^ ^31^ ^]^ glutamine‐dependent anaplerosis,^[^ ^32^ ^]^ and lipid metabolic abnormalities^[^ ^33^, ^34^ ^]^ contribute to tumor malignancy and radioresistance, metabolic target‐based strategies are ideal for optimizing cancer radiotherapy.^[^ ^35^ ^]^ In the present study, we focused on the regulatory effects of ACSLs on LCRS. First, we determined the expression profile of all *Acsls* in the Lewis lung carcinoma (LLC) cell line using RT‐qPCR. Only *Acsl6* was significantly upregulated at 24 and 48 h after exposure to 8 Gy IR (**Figure** **1**a; Figure S1a–d, Supporting Information). Consistently, analysis of the Cancer Genome Atlas (TCGA) indicated upregulation of *ACSL6* in patient tumors compared with that in paired adjacent normal lung tissue in both LUAD and LUSC (Figure 1b,c). The upregulation of *ACSL6* in tumors was also observed in differential expression analysis (Figure S3a,b, Supporting Information). Based on these results, we concluded that *ACSL6* is preferentially expressed in tumor tissues, and its expression is greatly upregulated by IR, and inferred that *ACSL6* may play an important role in the radiological response of LC. We next evaluated the relationship between *ACSL6* expression and the malignancy classification in detail based on the LUAD dataset in TCGA database (Figure S3c–e, Supporting Information). In particular, compared with that in tumor 1 (T1) group, the *ACSL6* levels in the T2, T3, and T4 groups were all significantly different, respectively (Figure S3c, Supporting Information). Further, the *ACSL6* levels of tumors between stage III/IV and stage I, and between M0 and M1 types, were also markedly different (Figure S3d,e, Supporting Information).

Next, we established LC cell lines stably expressing different levels of ACSL6 (Figure S1e–j, Supporting Information), and examined their radiosensitivity using a clonogenic assay. In contrast to that in wild‐type LLC cells (LLC WT), a dramatic drop in the surviving fraction was observed for LLC knockout (LLC KO) cells (Figure S2a,b, Supporting Information). In the H460 cell lines, a similar decrease in the surviving fraction was noted in the knockout (H460 KO) group whereas the trend was significantly reversed in the ACSL6‐overexpressing (H460 OE) group (Figure 1d; Figure S2d, Supporting Information), which was verified using a statistical comparison of the radiological parameters, such as surviving fraction at 2 Gy (SF2), α, and β (Figure 1e; Figure S2c, Supporting Information). Consistently, the results of the CCK8 assay also indicated that lack of ACSL6 led to a remarkable reduction in cell viability by IR, which was reversed by the restoration of ACSL6 function after 24, 48, and 72 h of IR treatment (Figure 1f). These LC cells were exposed to 8 Gy IR and then subjected to immunofluorescence (IF) staining for phosphorylated H2AX (γH2AX), which presents as foci and is a surrogate marker of DNA strand breaks, at the indicated time points, to further determine the altered radiosensitization of LLC and H460 cells. For all tested cell lines, foci per nucleus were the highest at 0.5 h after IR exposure and gradually decreased over time as DNA damage was cumulatively repaired. The KO group showed a lagged recovery of the IR‐induced increase in foci number, whereas the OE group showed the opposite trend (Figure 1g–i). Flow cytometry analysis revealed the deletion of ACSL6 promoted IR‐induced apoptosis, while the overexpression of ACSL6 was against it (Figure 1j–m). Collectively, these results suggest that ACSL6 participates in biological response of LC to IR and plays an indispensable role in maintaining the integrity of LC radioresistance.

### 2.2 Effects of ACSL6 on LCRS in Mice

**Figure 2:**
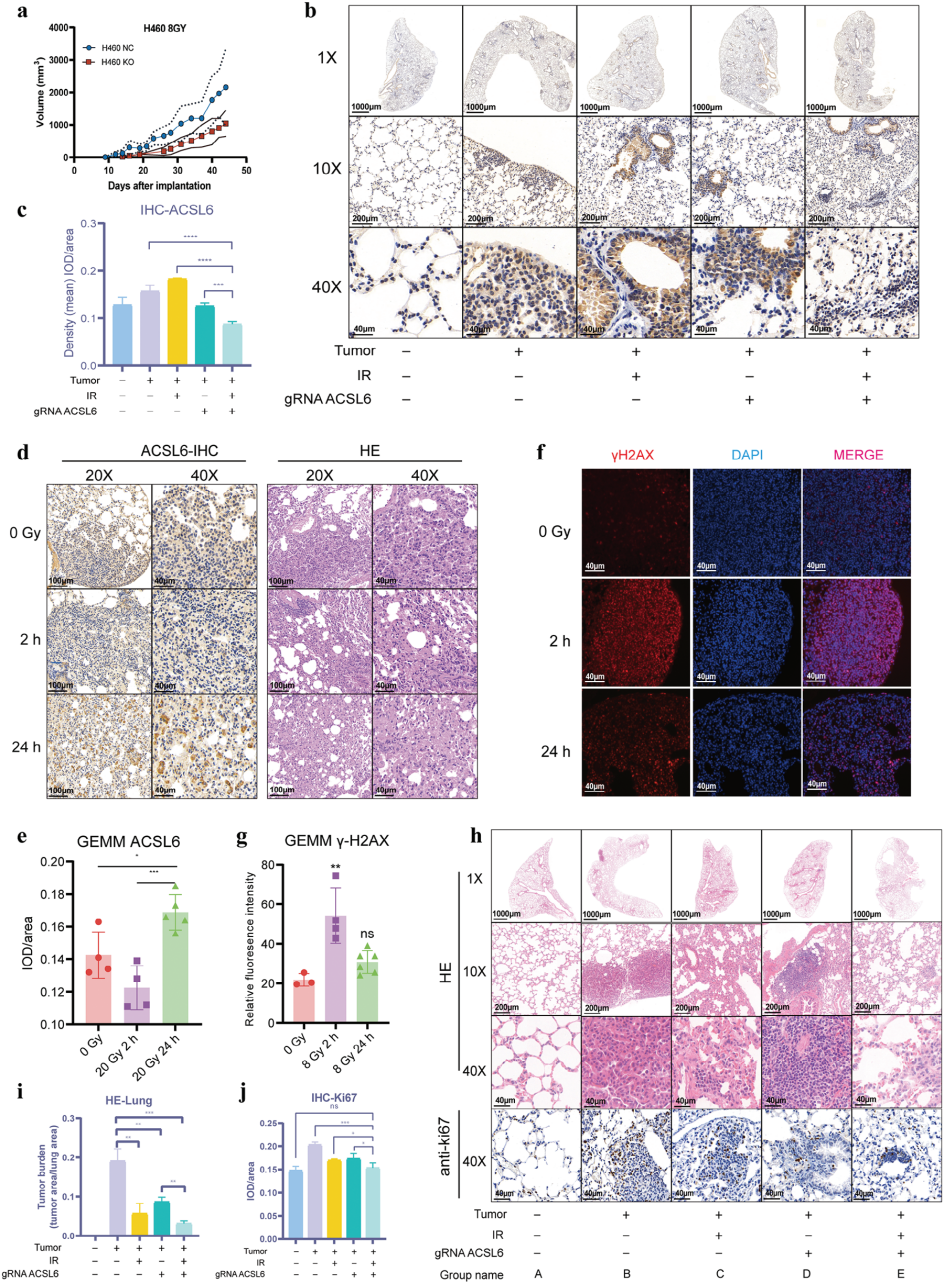
In vivo regulatory effects of ACSL6 on primary lung cancer radiosensitivity. a) Tumor growth curves for each group. Mice were randomly divided into two groups: NC + IR (*n* = 6) and KO + IR. At day 0, H460 NC and KO cells were inoculated subcutaneously into the right flank of mice. The first irradiation dose was delivered at day 7, when the mice developed a palpable mass (100–150 mm^3^) (*n* =  6 per group). b,c) Immunohistochemical (IHC) examination of tissues from Kras^G12D/+^ mice. Tissue sections were stained with the anti‐ACSL6 antibody. d) Representative IHC and hematoxylin and eosin (HE) staining images of the tumor sections are shown. The IR‐induced expression of ACSL6 in the tumors was verified using IHC staining. e) Bar graph showing the IOD/area values for ACSL6 signal for each group shown in panel (d). f) IR‐induced DNA damage dynamics in vivo. Representative images of γ‐H2AX immunofluorescence foci in tissue sections derived from the Kras^G12D/+^ mice sacrificed at the indicated times points after administration of 20 Gy IR are shown. g) Bar graph showing the relative fluorescence intensity of γ‐H2AX foci in images shown in panel (f). h) Representative images for HE and IHC staining of the tumor sections with the anti‐Ki67 antibody. i) Tumor burden (area ratios of the lung tumor/normal tissue) was calculated based on the HE‐stained sections represented in panel (h). j) Quantification of the Ki67‐positive areas (IOD/area ratio) based on anti‐Ki67 IHC of sections represented in panel (h). For all of the quantification based on the tissue sections, at least 10 fields were included for each group. Data are presented as mean ± SEM. The two‐tailed independent Student's *t*‐test was used for comparison between two groups. Ns, not significant, *p < 0.05, **p < 0.01, ***p < 0.001, ****p < 0.0001.

Based on in vitro and bioinformatics data, we posited that ACSL6 plays a crucial role in promoting radioresistance of LC. We tested this hypothesis using two in vivo models. First, we performed experiments on mice that were subcutaneously implanted with H460‐negative control (H460 NC) or H460 KO cells. After 8 Gy of local IR exposure, the tumor size in mice was measured continuously, and a tumor growth curve was plotted, which indicated that the tumor growth in the H460 KO group decreased significantly compared with that in the H460 NC group (**Figure** **2**a). In addition, we generated LSL‐K‐Ras^G12D/+^ transgenic mice, which were further induced into primary LC mouse models with or without the *ACSL6* KO manipulating (Figure S4a,b, Supporting Information). Eight weeks later, 20 Gy IR was locally applied on the chest of mice in groups C and E. After 5 weeks, the mice were sacrificed and their lungs were dissected. Immunohistochemical (IHC) analysis revealed a significant decrease in ACSL6 expression in groups D and E, confirming successful establishment of the ACSL6‐KO model (Figure 2b,c). Consistently, thyroid transcription factor‐1 (TTF‐1), a histological biomarker for indicating LC, was detected via IHC to confirm the primary LC generation. TTF‐1 levels were much higher in tumor‐like sites than in normal tissue sites, especially in Group B, indicating the reliability and fidelity of this genetically engineered mouse model (GEMM) (Figure S4c–g, Supporting Information). Next, we examined the regulation of LCRS by ACSL6 using the established GEMM. Hematoxylin and eosin (HE) and γH2AX staining at 2 and 24 h after 20 Gy local chest IR exposure within 1070 s showed considerably increased expression of ACSL6 at 24 h (Figure 2d,e), whereas the expression of γH2AX was the highest at 2 h after IR and was then reduced to a certain extent at 24 h (Figure 2f,g), which could be further verified partially using the HE analysis (Figure 2d). A more detailed analysis of HE‐stained sections indicated most serious parenchymal tumors in Group B, resulting in the highest tumor burden ratio (TBR; tumor area/lung area, Figure 2h,i). The calculated TBR for the five GEMM groups was ranked in the order B > D > C > E > A (Figure 2h,i). The Ki67 levels exhibited a similar trend, with brown staining signals, indicative of proliferation, being the strongest in Group B and weakest in Group E (Figure 2h,j). Taken together, these findings indicated that the lack of ACSL6 enhanced the LCRS in vivo and may ultimately improve the efficacy of radiotherapy in lung carcinoma.

### 2.3 Screening of the Mechanisms Underlying the ACSL6‐Mediated Regulation of LCRS

**Figure 3:**
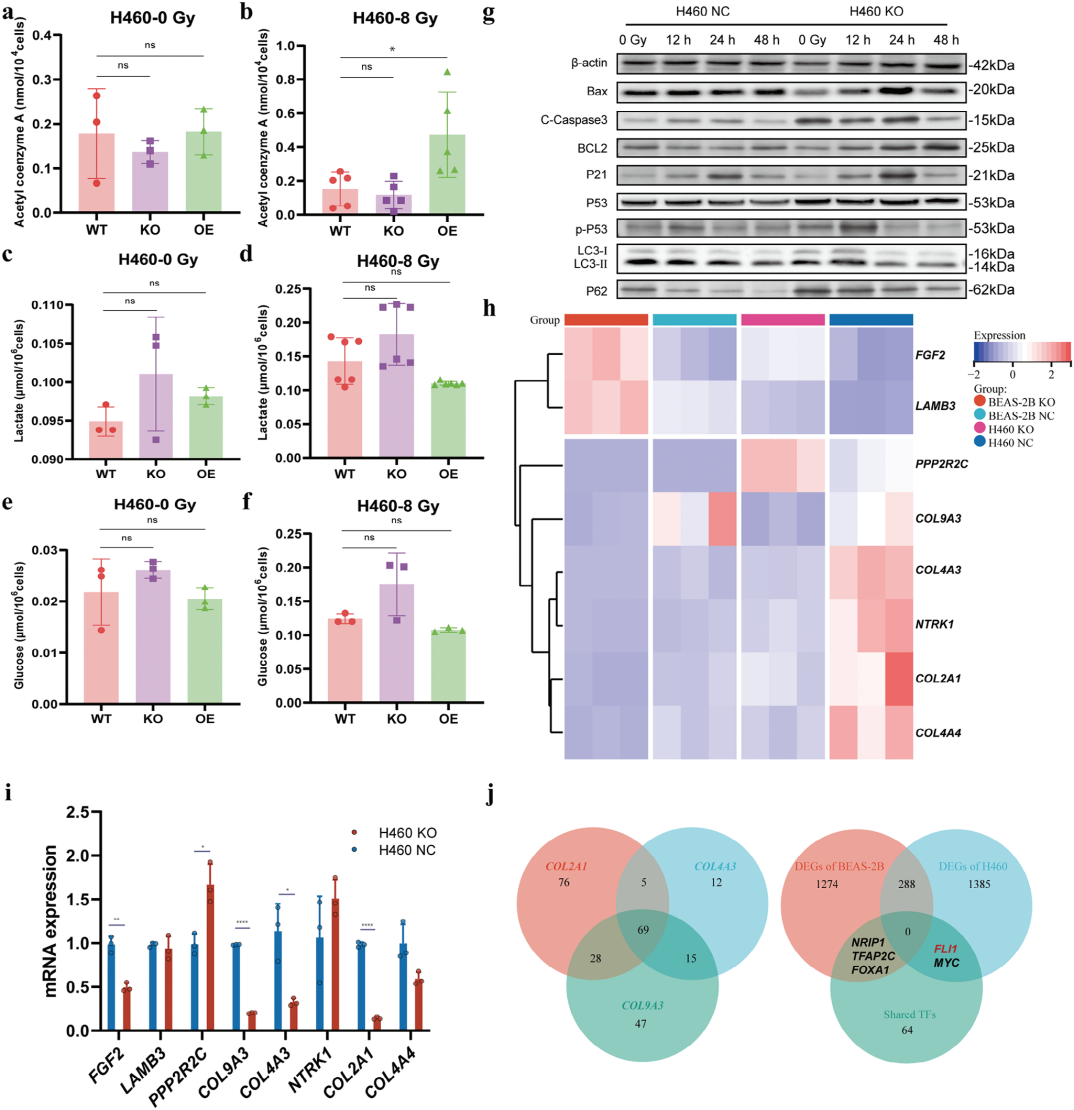
Global screening of the predominant molecular mechanisms underlying the regulation of lung cancer radiosensitivity by ACSL6. Levels of acetyl‐CoA (a,b), L‐lactate (c,d), and glucose (e,f) in H460 WT, KO, and OE cells before and after treatment with 8 Gy ionizing radiation (IR) (*n* = 3 per cell line). g) Expression of pivotal effectors relevant to the main radiological responses, as detected using western blot analysis at the indicated time points after IR treatment. h) Results of clustering analysis visualized using a heat map to identify the most differentially expressed genes in the different groups. i) Expression levels of *FGF2*, *LAMB3*, *PPP2R2C*, *COL9A3*, *COL4A3*, *NTRK1*, *COL2A1*, and *COL4A4*, as determined using RT‐qPCR (n = 4 per group). j) Venn diagram on the left showing the number of shared transcription factors (TFs) among COL9A3, COL4A3, and COL2A1. The upper half of the Venn diagram on the right shows the number of differentially expressed genes (DEGs); the three gene names mentioned are those of DEGs targeted by the screened TFs enriched by the left one. Data are presented as mean ± SEM. The Student's *t*‐test was used for comparison between two groups. Ns, not significant, *p < 0.05, **p < 0.01, ****p < 0.0001.

As ACSL6 is crucial in the activation of FAs, which is necessary for energy metabolism, we first investigated the energy metabolism remodeling to elucidate the mechanism of LCRS regulation by ACSL6.^[^ ^22^ ^]^ Alterations in ACSL6 expression slightly affected the acetyl‐CoA levels in H460 cells, except for the comparison between H460 KO and H460 OE after IR treatment (**Figure** **3**a,b). What should be noted is that the H460 KO group did not exhibit significant differences from H460 WT group, whether IR‐treated or not, which is not consistent with the radiosensitized phenotype of the ACSK6 KO cells. With regard to glucose metabolism, ACSL6 knockout also slightly upregulated the glucose and lactate levels in H460 cells with or without IR treatment (Figure 3c–f). These data indicated that the regulation of LCRS by ACSL6 did not mainly rely on its intrinsic ability to regulate energy metabolism, because the loss of ACSL6 also induced marked radiosensitization of LC cells, but no obvious alterations in energy metabolism were detected (Figure 3a–f). Next, we examined other cancer characteristics closely related to the radiosensitization of cancer cells, such as reactive oxygen species (ROS) stress, cell cycle, migration, senescence, apoptotic transduction, and autophagy at different time points (12, 24, and 48 h) after 8 Gy IR treatment. ROS levels were evaluated using IF and flow cytometry employing the DCFH‐DA marker. The lack of ACSL6 markedly upregulated the ROS levels, especially after IR exposure (Figure S5b–h; Figure S8a,e, Supporting Information). Additionally, cell cycle analysis revealed that IR exposure resulted in the accumulation of significantly more ACSL6 KO cells in the G1 phase compared with that in the case of WT cells (Figures S6 and S8b, Supporting Information). The loss of ACSL6 rendered a significantly higher percentage of senescent cells 48 h after IR exposure (Figures S7a and S8c, Supporting Information). Moreover, the wound‐healing assay revealed impairment of the migratory ability of H460‐KO cells exposed to 8 Gy IR (Figures S7b and S8d, Supporting Information). Next, we determined the levels of several classical markers of apoptosis, cell cycle, ROS stress, and autophagy using western blot analysis. The expression of Bax, C‐caspase 3, P21, and P‐P53 was markedly increased whereas the LC3II/LC3I ratio was decreased in ACSL6‐KO cells after exposure to 8 Gy IR. The P62 levels were increased after knockout of *ACSL6* in H460 cells, and decreased in a time‐dependent manner after IR exposure in both of the cell lines (Figure 3g; Figure S8f–m, Supporting Information). These results imply that the loss of ACSL6 promotes IR‐induced apoptosis and phosphorylation of P53, interferes with IR‐remodeled cell cycle distribution, and hinders IR‐induced autophagy.^[^ ^36^ ^]^ Collectively, the results presented in this section indicated the potential involvement of several pathways for the regulation of LCRS by ACSL6 and paved the way for a further in‐depth investigation of a specific mechanism.

To further explore the specific mechanism of ACSL6‐mediated regulation of LCRS, we conducted a transcriptomic analysis. We identified the differentially expressed genes (DEGs) between ACSL6 NC and ACSL6 KO cells (H460 and BEAS 2B) via RNA sequencing (Figure S8n,o, Supporting Information). A hub of 78 DEGs was downregulated, whereas another 88 DEGs were upregulated in the ACSL6 KO groups in both the cell lines (Figure S8p, Supporting Information), implying that the fundamental and conserved pivotal molecular effectors responding to the intervention of ACSL6 expression were most likely within these intersectional genes. The Kyoto encyclopedia of genes and genomes (KEGG) enrichment analysis was performed to identify the most relevant pathways. Although over 20 biological pathways were significantly enriched, we focused on the hsa04151: PI3K‐Akt signaling pathway based on careful considerations including their level of importance to radiotherapy and the accessibility to the specific robust biological pathway (Figure S8q, Supporting Information). We analyzed the expression of all the relevant eight DEGs within the PI3K‐Akt signaling pathway and visualized it as a heatmap. Genes including *COL4A3*, *COL2A1*, *COL4A4*, and *NTRK1* were significantly downregulated in H460 KO cells but not in BEAS 2B KO cells. In contrast, genes such as *FGF2* and *LAMB3* were only markedly upregulated in BEAS 2B KO cells (Figure 3h). Particularly, the expression of *PPP2R2C* was upregulated in H460 KO cells, but *COL9A3* was substantially downregulated in both the ACSL6 KO cell lines (Figure 3h). Subsequently, the expression levels of all the above eight genes in H460 KO/NC cells were verified using RT‐qPCR; the changes in the expression of *COLs (COL9A3, COL4A3, COL2A1)* were consistent with the previous transcriptome analysis data (Figure 3i). These results indicated that ACSL6 regulates the levels of *COLs* mRNAs in LC cells. Transcription factors (TFs) common to these genes were predicted using the hTF target database (http://bioinfo.life.hust.edu.cn/hTFtarget#!/) (Figure S9a, Supporting Information), showing that *COL2A1*, *COL4A3*, and *COL9A3* shared 69 TFs (Figure 3j). Finally, the followed Venn diagram suggested that NRIP1, TFAP2C, and FOXA1 in BEAS 2B cells and FLI1 and MYC in H460 cells were deemed to play probable roles in mediating the effect of ACSL6 (Figure 3j).

### 2.4 FLI‐1 Transcriptionally Regulates COLs in an ACSL6‐Dependent Manner

**Figure 4:**
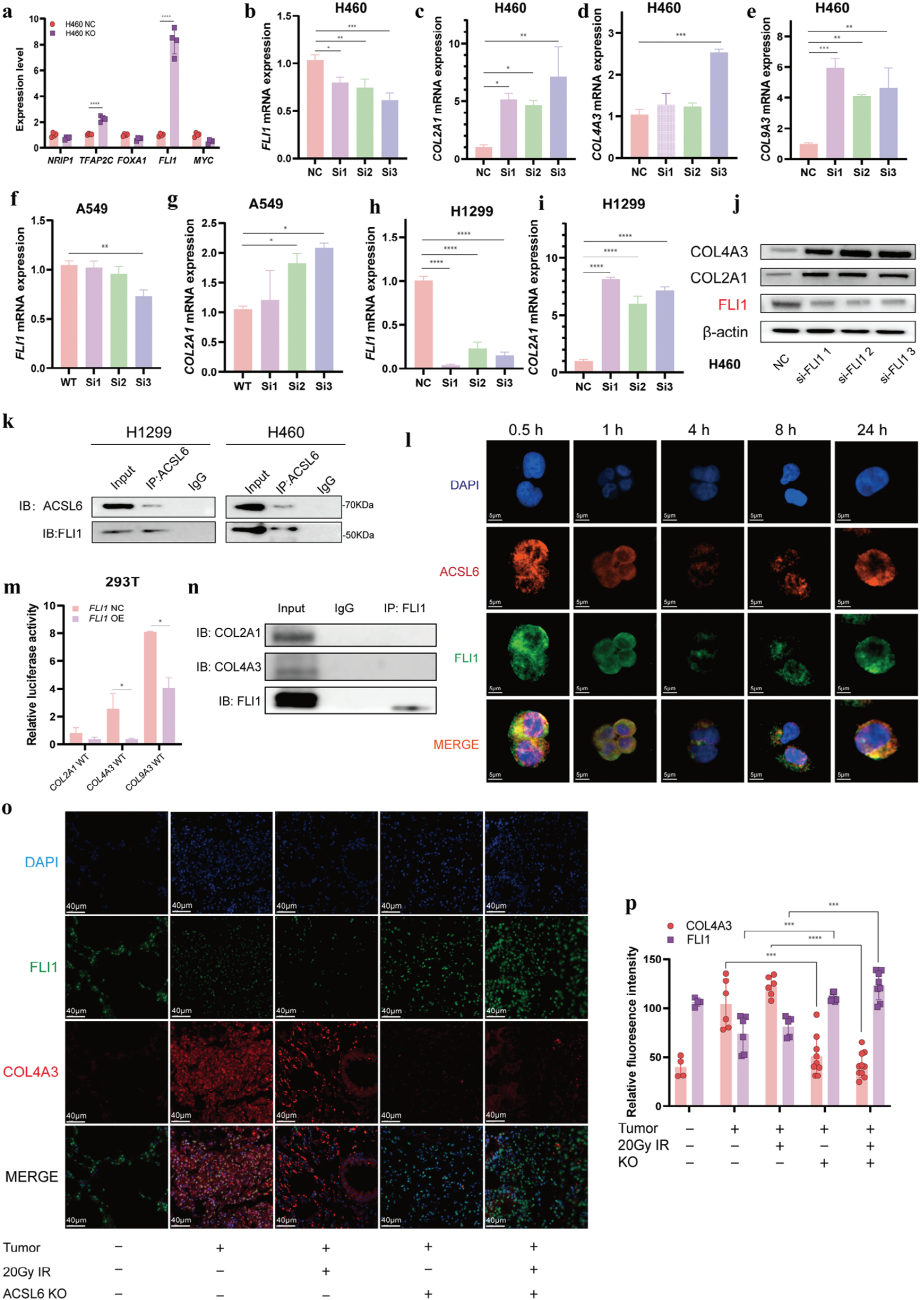
The ACSL6‐engaged FLI1 transcriptionally regulates the expression of COLs in vitro and in vivo. a Expression levels of *NRIP1*, *TFAP2C*, *FOXA1*, *FLI1*, and *MYC* in H460 NC and KO cells, as determined using RT‐qPCR (*n* = 4 per group). b–e Expression levels of *FLI1*, *COL2A1*, *COL4A3*, and COL9A3 after knock down of FLI1 in H460 cells, as determined using RT‐qPCR (*n* = 4 per group). f–i) Expression levels of *COL2A1* after knock down of FLI1 in A549 and H1299 cells, as determined using RT‐qPCR (*n* = 4 per group). j) Protein levels of COLs after knock down of FLI1 in H460 cells, as determined using western blot analysis. k) Interaction between ACSL6 and FLI1 in cell lines including WT and ACSL6 overexpressing H1299 (left) and WT and ACSL6 overexpressing H460 (right), as determined using coimmunoprecipitation (Co‐IP) assay. l) Assessment of the spatial and temporal relationship between ACSL6 and FLI1 in H460 cells, as determined using immunofluorescence analysis(Integration times = 2250 ms, camera gain = 13). m) Evaluation of the direct binding between FLI1 and the promoters of COLs, as determined using the dual‐luciferase reported assay. The WT target sequences of COL2A1, COL4A3, and COL9A3 were co‐transfected into 293T cell lines harboring negative control plasmid or FLI1 plasmid‐high (*n* = 3 per group). n) Representative bands visualized in the Co‐IP assay showing the direct interaction between FLI1 and COLs. o) Representative immunofluorescence staining images of FLI1 and COL4A3 from the GEMM tissue sections. p) Quantification and comparison of the relative fluorescence intensity of FLI1 and COL4A3 for images presented in panel o (at least 10 fields were included for each group). Data are presented as mean ± SEM. The two‐tailed independent Student's *t*‐test was used for comparison between two groups. *p < 0.05, **p < 0.01, ***p < 0.001, ****p < 0.0001.

Next, we validate the expression of the screened TFs using RT‐qPCR. FLI1 was found to be the most prominent TF, with a significant difference in expression between the NC and ACSL6‐KO H460 groups (**Figure** **4**a). Furthermore, the lack of ACSL6 significantly upregulated *FLI1* expression and downregulated *COL* expression (Figures 3i and 4a). Thus, we assumed that the loss of ACSL6 suppressed the expression of *COLs* through positive regulation of their shared TF, FLI1. To test this hypothesis, we performed RT‐qPCR using different FLI1‐knockdown (FLI1‐KD) cells. The FLI1‐KD cells were successfully established (Figure 4b,f,h). In FLI1‐KD H460 cells, the mRNA levels of all *COLs* were markedly increased. Moreover, *COL2A1* mRNA levels were consistently increased in FLI1‐KD cells compared with those in NC cells across all three cell lines (Figure 4b–i), and the levels of COL2A1 and COL4A3 proteins were consistently upregulated by FLI1 knockdown (Figure 4j). For further validating the regulatory role of FLI1 as a TF for COLs, we performed a coimmunoprecipitation (Co‐IP) experiment to detect the interaction between ACSL6 and FLI1 and observed a direct protein–protein interaction between them in both H460 and H1299 cells (Figure 4k). This was verified using an IF assay to visually detect the spatial and temporal relationship between ACSL6 and FLI1 within H460 cells. The confocal images showed that the level of colocalization between ACSL6 and FLI1 after IR was observed instantaneously, and remained increased for 8 h. After 24 h, the expression of ACSL6 and FLI1 was faded, with loss of colocalization (Figure 4l). We also performed a dual‐luciferase reporter assay (DLR) to verify whether FLI1 binds to the promoters of *COL2A1*, *COL4A3*, and *COL9A3*. FLI1 directly interacted with the promoters of *COL2A1*, *COL4A3*, and *COL9A3* and inhibited the luciferase activity (Figure 4m). Moreover, no direct interactions between FLI1 and COLs were noted in Co‐IP, which further proved that FLI1 regulates COLs in a transcriptional manner (Figure 4n). IF analysis of lung sections of GEMM showed that the loss of ACSL6 led to an increase in FLI1 expression, but resulted in a decrease in COL4A3 expression in Group D, which was more pronounced in Group E (Figure 4o,p). These results indicate that ACSL6 regulates COLs by targeting their TF FLI1 both and in vitro in vivo.

To further decipher the mechanism involved in the regulation of LCRS by ACSL6‐mediated modulation of COL expression, we performed a metabolomic analysis 24 and 48 h after exposure to 8 Gy IR. The principal component analysis and partial least squares‐discriminant analysis data exhibited good quality control, suggesting that there was no risk of overfitting of data (Figures S10a and S11a, Supporting Information). The super‐class clustering analysis using the Human Metabolome Database revealed that lipids and lipid‐like molecules were the most regulated metabolites (Figure S10b, Supporting Information). The KEGG enrichment analysis of the regulated compounds (Figure S11b,c, Supporting Information) indicated that the autophagy‐animal pathway was the most dramatically affected, with the strongest enrichment factor among the key specific mechanistic radiobiological information orienting classes (Figures S10c and S12–S14, Supporting Information). Consistent with this, our previous mechanism screening data suggested that ACSL6 could markedly regulate critical autophagy molecular mediators, including BCL2, LC3, and P62 (Figure S8g,l,m, Supporting Information). Based on these results and comprehensive analysis, we assumed that the regulation of LCRS by ACSL6 depended on autophagy as the terminal cellular pathway considering the well‐documented role of IR‐induced autophagy in radioresistance.

### 2.5 ACSL6 Regulates LCRS by Tuning the FLI1‐COL‐Autophagy Pathway

**Figure 5:**
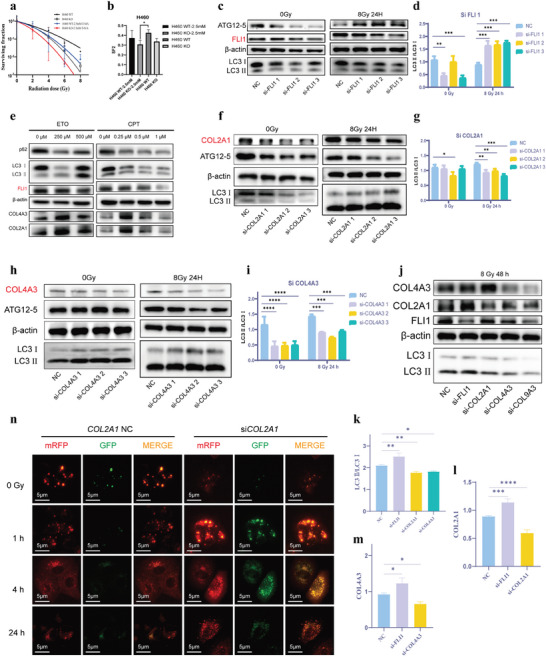
Investigation of the regulation of COLs on the ionizing radiation (IR)‐induced cellular autophagy. a,b) Evaluation of the effects of 3‐MA (2.5 mm) on the regulation of lung cancer radiosensitivity by ACSL6 based on clonogenic curves and SF2 comparisons (*n* = 3 for each replicate). c) Expression of FLI1, ATG12‐5, LC3II, and LC3I in H460 NC and FLI1‐KD cell lines (H460 cells were treated with different siRNAs: FLI1 NC, si‐FLI1 1, si‐FLI1 2, and si‐FLI1 3) following sham or IR treatment, as determined using western blot analysis. d) Quantification and comparison of the LC3II/LC3I ratio based on the intensity of bands in the immunoblots shown in panel (c) (*n* = 3 per group). e) Expression of COLs and autophagy flux proteins, as detected using western blot analysis after incubation of cells with different concentrations of FLI1 inhibitors ETO and CPT. f) Western blot analysis of COL2A1 and autophagy markers ATG12‐5, LC3II, and LC3I in NC and COL2A1‐KD cell lines (H460 cells were treated with different siRNAs: COL2A1 NC, si‐COL2A1 1, si‐COL2A1 2, and si‐COL2A1 3) following sham or IR treatment. g) Quantification and comparison of the LC3II/LC3I ratio based on the intensity of bands in the immunoblots shown in panel (f) (*n* = 3 per group). h) Western blot analysis of COL4A3 and autophagy markers ATG12‐5, LC3II, and LC3I in NC and COL4A3‐KD cell lines (H460 cells were treated with different siRNAs: COL4A3 NC, si‐COL4A3 1, si‐COL4A3 2, si‐COL4A3 3) following sham or IR treatment. i) Quantification and comparison of the LC3II/LC3I ratio based on the intensity of bands in the immunoblots shown in panel (h) (*n* = 3 per group). j) Representative images of immunoblots showing IR‐stimulated levels of FLI1, COL2A1, COL4A3, LC3II, and LC3I in H460 NC, siFLI1, siCOL2A1, siCOL4A3, and siCOL9A3 cell lines. k–m) Bar graphs showing the levels of COL2A1 and COL4A3, and the LC3II/LC3I ratio based on the intensity of bands in the immunoblots shown in panel (j) (*n* = 3 per group). n) Representative images of H460‐RFP‐GFP‐hLC3 cells under 1, 4, and 24 h after IR. Data are presented as mean ± SEM. The two‐tailed independent Student's *t*‐test was used for comparison between two groups. *p < 0.05, **p < 0.01, ***p < 0.001, ****p < 0.0001.

To test our hypothesis that ACSL6 regulated LCRS by tuning the FLI1‐COL‐autophagy axis, we determined the changes in autophagic flux after manipulating the expression of FLI1 and COLs. The clonogenic assay clearly demonstrated that the ACSL6 knockout combined with autophagy inhibition resulted in the best radiosensitizing effect (**Figure** **5**a,b). Next, we investigated the protein levels of autophagic molecular pivots and observed that knockdown of FLI1 increased the LC3II/LC3I ratio and ATG12‐5 levels (Figure 5c,d; Figure S15e,f, Supporting Information). Similarly, the COL2A1 and COL4A3 levels were markedly increased in FLI1‐KD after IR exposure (Figure S15a–d, Supporting Information). Consistently, after incubation with the FLI1 inhibitors, etoposide (ETO), and camptothecin (CPT), the LC3II/LC3I ratio was significantly increased and the P62 level was significantly decreased, accompanied by an increase in the levels of COLs (Figure 5e). After knocking down of COL2A1 and COL4A3, the evaluation of the autophagy changes in LC cells before and after IR exposure showed markedly downregulated levels of both ATG12‐5 and the LC3II/LC3I ratio (Figure 5f–i; Figure S15g–j, Supporting Information). Moreover, after selecting and applying the siRNAs with the highest KD efficiency among si‐FLI1s, si‐COL2A1s, and si‐COL4A3s, we found the same trend at 48 h after IR exposure (Figure 5j–m). Subsequently, we construct the lentivirus vector to germinate H460 cells producing LC3‐GFP‐mRFP to indicate the autophagic flux. In absence of IR, the siCOL2A1 obviously decreased the expression of LC3, which was consistent with our previous results. Intriguingly, after exposure to IR, both of NC and siCOL2A1 initiated the process of autophagy with the expression of LC3 markedly elevated. However, the autophagic flux in the siCOL2A1 group was at a standstill while the GFP intensity was eradicated because of the integration of autophagosome and lysosome in the NC group. One hour after IR, the GFP signal continuously reduced because the acidic environment of autolysosomes degrades GFP but not mRFP in NC group. In contrast, although the GFP and mRFP signal both increased indicating the synthesis of LC3 was enhanced, the GFP intensity remained at a high level, which suggested the integration of autophagosome and lysosome was interrupted (Figure 5n). Overall, these results indicated that FLI1‐induced suppression of COLs could further lead to a decline in IR‐induced autophagy, ultimately resulting in cellular death and radiosensitization. Thus, ACSL6 modulates LCRS by adjusting the FLI1‐COL‐autophagy pathway.

### 2.6 ACSL6 Can be a Potential Novel Therapeutic Target for Refining LCRS

**Figure 6:**
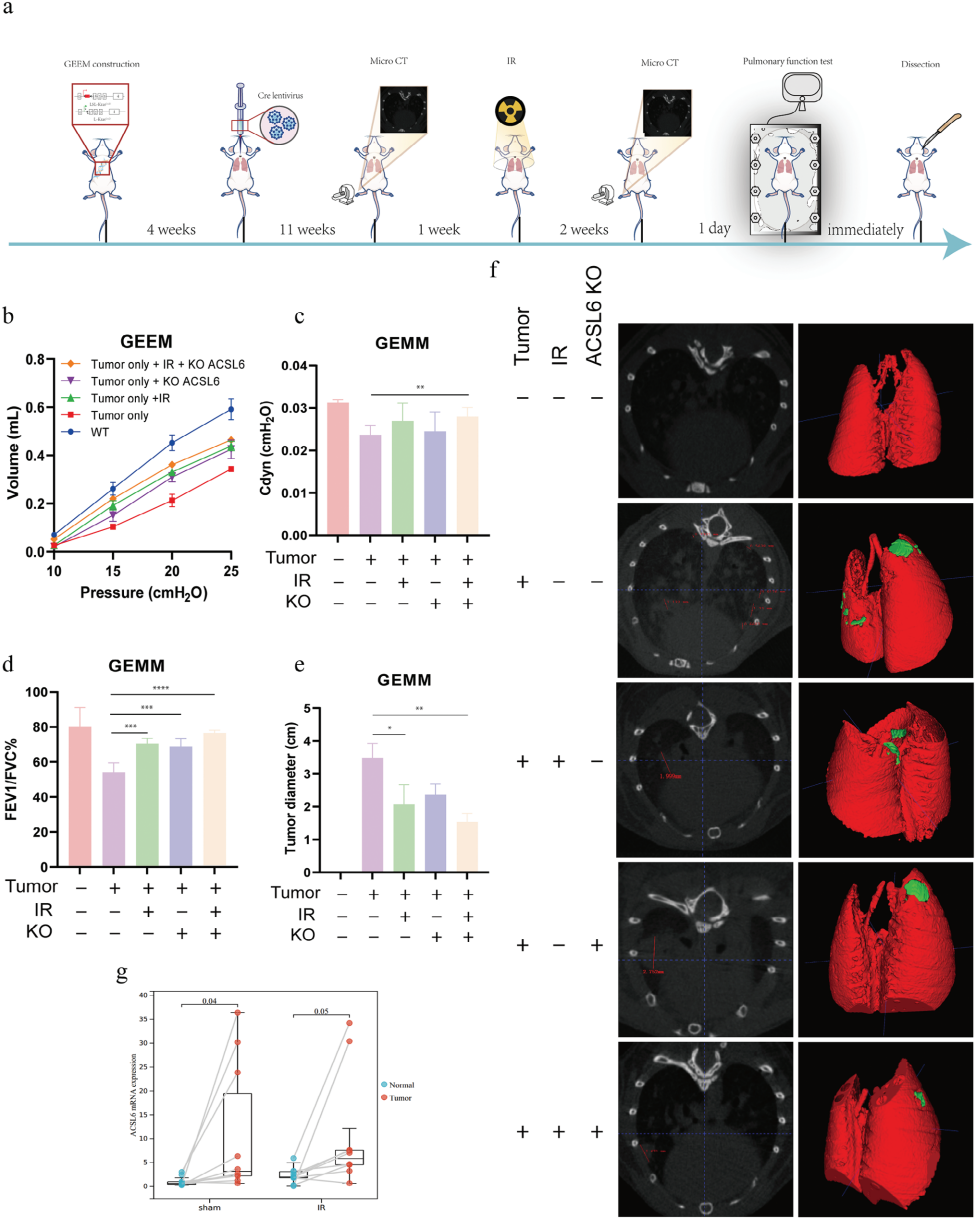
Assessment of the clinical potential of targeting ACSL6 as an ameliorative strategy for improving the radiotherapy of lung cancer. a) Schematic of the workflow for in vivo experiment. b–d) Quantitative comparison of the pulmonary function test indicators, including Cdyn, FEV1/FVC%, and pressure‐volume curves. e) Bar graph shows the mean lung tumor diameter for each group measured using micro‐computed tomography (Micro‐CT). f) Representative images of Micro‐CT scans and 3D model fitting of the mouse lung using ITK‐SNAP. g) Changes in the expression of *ACSL6* after sham or ionizing radiation (IR) treatment in the lung tumor and adjoining normal tissues from patients, as assessed using RT‐qPCR (*n* = 20 per group). For GEMM experiments, WT: *n* = 4; Tumor only: *n* = 5; Tumor only + IR: *n* = 5; Tumor only + KO: *n* = 6; Tumor only + IR + KO: *n* = 7. Data are presented as mean ± SEM. The two‐tailed independent Student's *t*‐test was used for comparison between two groups. *p < 0.05, **p < 0.01, ***p < 0.001, ****p < 0.0001.

To evaluate the clinical prospects of ACSL6, we conducted a pulmonary function test (PFT) reflecting the respiratory reserve as well as the micro‐computed tomography (Micro‐CT) monitoring the tumor volume in GEMM (**Figure** **6**a). Both IR and loss of ACSL6 were effective in restoring the key PFT indicators (Cdyn, FEV1/FVC%, and P‐V curve) that were suppressed by IR, with the combination treatment performing the best. The performance ranking of the lung function among the five groups was as follows: A > E > C > D > B (Figure 6b–d). The Micro‐CT data indicated that the tumors in Group B had the longest diameter, which was markedly decreased by loss of ACSL6 and IR alone and decreased to a greater extent by their combination (Figure 6e,f). Furthermore, RT‐qPCR analysis indicated a significant increase in the expression of *ACSL6* in human lung tumor samples after IR exposure, which was consistent with the previous findings (Figure 6g). Collectively, this preclinical evidence, based on the examination of a primary lung carcinoma model and clinical samples, suggested that ACSL6 can be a potential clinical adjuvant candidate target which can provide novel strategy with better radiotherapeutic effects.

## 3 Discussion

**Figure 7:**
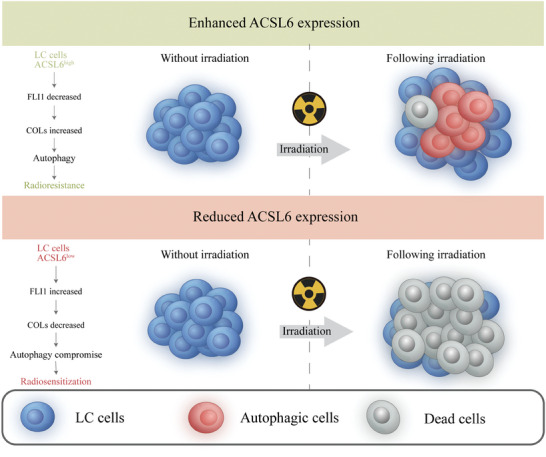
Schematic illustrating the ACSL6‐FLI1‐COL‐Autophagy axis‐mediated cellular adaptation to radiotherapy stress in lung cancer (LC). Independent of its classical enzymatic effects on lipid synthesis, ACSL6 plays an essential role in the development of LC radioresistance by directly reciprocal targeting of FLI1. Thereafter, FLI1 suppresses the ionizing radiation (IR)‐induced autophagic flux by transcriptionally modulating the COLs, leading to the regulation of lung cancer radiosensitivity.

Although many attractive advances have been made regarding LCRS, much remains to be done to further improve the radiotherapy of LC. In this study, we discovered a unique radiological response of ACSL6 in LC, which plays an important role in the progression of malignancy. Based on these findings, we elucidated the role of IR‐induced ACSL6 in conferring radioresistance to LC. Additionally, we revealed that this radiosensitization process was promoted by the FLI1‐COL‐autophagy pathway (**Figure** **7**). Thus, blocking the ACSL6‐FLI1‐COL axis may suppress the development of LC radioresistance, which is equipped with autophagy‐sustained self‐rescue against radiotherapy.

FA metabolism is highly relevant to malignant phenotypes of LC, including tumorigenesis and development.^[^ ^37^ ^]^ The ACSL family, comprising PPARγ‐targeted genes, which mediates lipid metabolism and regulates the caloric absorption, is a crucial enzyme family in FA metabolism and exhibits numerous connections to tumorigenesis and tumor progression.^[^ ^38^ ^]^ For example, ACSL3 is instrumental in the generation of mutant Kras LC tumorigenesis^[^ ^39^ ^]^ and its knockdown reduces the growth of LC cells.^[^ ^40^ ^]^ Also, ACSL4 affects the steroid hormone‐sensitivity in prostate and breast cancer.^[^ ^41^ ^]^ ACSL6 is abundant in the plasma membrane and exhibits potent activity for C16‐C20 saturated and polyunsaturated fatty acids.^[^ ^42^ ^]^ Recently, a bioinformatics study showed that the expression of ACSL6 was decreased in some types of cancers but was increased in others.^[^ ^43^ ^]^ Considering the accumulating evidence that even the same member of ACSLs may play distinct roles in different types of cancer, clarifying the exact role of ACSL6 in modulating the progression and therapeutic sensitivity of LC required robust experimental examination.

IR treatment alters several biological behaviors, such as migration, transformation, and metabolism, most of which contribute to the development of malignancy and therapeutic resistance.^[^ ^44^, ^45^ ^]^ IR‐induced changes in the expression of specific genes are critical to the evolution of acquired malignant phenotypes favored by the initiation of signal transduction cascades for maintaining cellular homeostasis.^[^ ^46^, ^47^ ^]^ Because we observed the radiation response of ACSL6 in LC cells and further noticed its crucial role in promoting LC progression, we investigated its effects on radiosensitivity in detail. The results of the clonogenic survival assay, a well‐established classical indicator for assessing radiosensitivity, showed that the lack of ACSL6 increased the radiosensitivity of LC cells (Figure 1d,e; Figure S2, Supporting Information). Consistently, in vitro data regarding several main cellular processes affecting radiosensitivity collectively demonstrated that ACSL6 is indispensable for maintaining resistance to IR in LC (Figure 1f–m).^[^ ^48^ ^]^ Cancer cells usually employ increased autophagy as a survival strategy against radiotherapy, leading to the development of therapeutic resistance.^[^ ^49^, ^50^, ^51^, ^52^ ^]^ Additionally, autophagy can either regulate or is regulated by DNA damage‐processing signals, thereby, markedly affecting radiosensitivity.^[^ ^53^, ^54^ ^]^ These results imply that ACSL6 may influence LCRS by modulating autophagy and apoptosis, which has also been confirmed through experiments on two in vivo LC models, with the xenograft tumor‐bearing model simulating human LC cells and GEMM closely simulating clinical radiotherapy scenarios. For example, in the combined investigation based on the HE analysis and PFT findings, we observed that Group B had the lowest Cdyn value (0.023) but the most severe parenchymal tumors, resulting in the highest TBR, implying that the severe tumor lesions in Group B had markedly impaired lung compliance (Figures 2h,i and 6c). In contrast, interventions, including the removal of ACSL6, IR, and a combination of the both mitigated TBR, with the combination treatment exhibiting the best results (Figure 2h,i). Taken together, these findings indicate that ACSL6 modulates LCRS, and that the underlying mechanism may be related to autophagy and apoptosis.

As mentioned above, the ACSLs are among the most critical rate‐limiting enzymes for FA utilization. Therefore, we had reasons to first attempt to explore the possible mechanisms relevant to energy metabolism centered on lipid and glucose flux. Unexpectedly, our data indicated that ACSL6 affects LCRS by means other than its known capability to regulate energy metabolism (Figure 3a–f). Thus, after further detecting the pivotal molecular effectors along the canonical radio‐biological pathways, we found that ACSL6 KO rendered LC cells more apoptotic, senescent, and ROS‐prone, with more cells accumulating in the G1 phase after IR exposure (Figure 3g; Figures S5–S7, Supporting Information). To the best of our knowledge, ROS can upregulate autophagy, and their levels are also reduced by the latter; the underlying mechanisms may involve signaling pathways, including the ROS‐NRF2‐P62‐autophagy and P62 delivery pathway.^[^ ^55^, ^56^ ^]^ Consistently, our data suggest that ACSL6 KO results in high levels of ROS but decreased autophagy flux, indicating that the reduction in autophagy results in increased release of ROS, which act as typical cell death stimuli. As a central regulator of the crosstalk between apoptosis and autophagy, BCL‐2 is well characterized as an inhibitor of both apoptosis, through its interaction with BAX/BAK, and autophagy, through its binding to Beclin‐1.^[^ ^57^, ^58^ ^]^ Interestingly, under some circumstances, BCL‐2 can also function as an inducer of apoptosis.^[^ ^59^, ^60^, ^61^, ^62^ ^]^ Based on the results presented in Figure 4h and Figure S8f–h (Supporting Information), we can conclude that the lack of ACSL6 upregulates BCL‐2, BAX, and cleaved Caspase3, and decreases autophagy flux levels manifested by changes in LC3 and P62. Considering these results, we infer that BCL‐2 may be vital for inhibiting IR‐induced autophagy and contributing to apoptosis. Autophagy activates and inhibits cellular senescence under different cellular settings.^[^ ^63^ ^]^ Collectively, these results provide important mechanistic insights centered on autophagy through which ACSL6 modulates LCRS.

To clarify the underlying mechanism, we conducted transcriptomic, metabolomic, and bioinformatics analyses to identify the most likely cellular processes (Figure 3h–j; Figures S10–S14, Supporting Information), and confirmed the results using laboratory experiments, such as RT‐qPCR, Co‐IP, and DLR assays. As elaborated above, the ACSL6‐FLI1‐COL‐autophagy signal transduction pathway was found to be the mechanism by which ACSL6 affects LCRS. Notably, Co‐IP results indicated that ACSL6 directly interacts with FLI1, and the DLR assay showed that FLI1 binds to the promoters of *COLs*. These results indicate that ACSL6 directly regulates COLs through molecular interactions, which, in turn, supports the IR‐induced antistress autophagy process. Recently, several studies have shown that COLs play an important role in regulating the autophagic turnover. Using a COL6‐null mouse model and fibroblasts/primary neural cultures, COL6 ablation was shown to impair autophagy.^[^ ^64^, ^65^, ^66^, ^67^, ^68^ ^]^ Similarly, supplementation with denatured COL1 reactivated autophagy and decreased apoptosis in fibroblasts.^[^ ^69^ ^]^ Another clinical study revealed that diseases caused by abnormalities in collagen metabolism, such as POP, impaired the autophagy flux.^[^ ^70^ ^]^ In line with previous studies, in the present study, we found that COLs positively regulate IR‐induced autophagy, and FLI1‐inhibited COLs result in an evident downregulation of IR‐induced cytoprotective autophagy. Thus, these findings extend our knowledge of the mutual relationship between autophagy and collagen under cancerous conditions.

Although no detailed investigation on how suppression of autophagy boosts IR‐induced cellular catastrophe is provided here, we can still draw some clues from our cell cycle evaluation for a better understanding of this phenomenon. We found that ACSL6‐KO LC cells had a much higher population in the G1 phase than the WT cells (Figure S6, Supporting Information), and the autophagy flux appeared to be inhibited in ACSL6 KO cells (Figure 3). Autophagy of mitotic arrested cells acts as a protective mechanism by preventing the unintended loss of organelles and chromosomes during mitosis^[^ ^71^ ^]^ this important protective mechanism was lost by the resulting deficiency in autophagy after KO of ACSL6 in the present study. Interestingly, CDK4/6 and CDK2 have been identified as key promoters of the G1/S transition and have been reported to inhibit autophagy.^[^ ^72^, ^73^, ^74^ ^]^ P21, which contributes to G1 arrest, is an inducer of autophagy.^[^ ^75^, ^76^ ^]^ Therefore, we deduce that the changes in autophagy are less likely a major consequence of the modulation of these cell cycle regulators, as our data indicate that in LC cells, ACSL6 KO is accompanied by P53‐P21–sustained G1 arrest but results in the inhibition of autophagy (Figure 3g; Figure S6, Supporting Information). ACSL6 KO disturbs the correlation between cell cycle rhythm and autophagy flux and hinders the IR‐initiated G1 arrest‐induced autophagic cell protective mechanism; however, a fine delineation of this intriguing relationship is warranted in the future.

Autophagy, one of the most versatile mechanisms, plays a crucial role during radiotherapy, with two opposing functions, cytoprotective and cytotoxic, in response to IR.^[^ ^77^ ^]^ During the tumor initiation stages, autophagy helps clear damaged mitochondria and aberrant protein aggregates that produce ROS to prevent carcinogenesis; however, after the tumor has formed, autophagy becomes a prosurvival mechanism against therapeutic stress, including IR.^[^ ^78^ ^]^ A growing body of evidence demonstrates that inhibition of autophagy, or conversion of autophagy from a cytoprotective to a cytostatic role, mediates radiosensitization of LC and breast cancer.^[^ ^79^, ^80^, ^81^ ^]^ By establishing an animal model relevant to clinical practice and adopting reliable measurements, including Micro‐CT monitoring (Figure 6e,f), we showed that the ACSL6‐tuned autophagy affects the LC response to radiotherapy, and shows good prospects for clinical application.

## 4 Outlook

In this study, we found that IR‐elevated ACSL6 expression modulates LCRS by tuning the FLI1‐COL‐autophagy pathway. By targeting and decreasing ACSL6, the activated molecular signal transduction chain inhibits the autophagic flux, which compromises its cytoprotective function against therapeutic stress. ACSL6 has been acknowledged for its substantial effect on FA metabolism in the brain and its involvement in facilitating spermatogenesis, and similar mechanisms is noteworthy for broadening our understanding of its effect in the control of cancer, particularly with respect to LCRS. Potential novel molecular targets along the ACSL6‐dominated FLI1‐COL‐autophagy pathway, presented in the mechanistic diagram, need to be further explored and developed. Moreover, we will continue to explore the more concrete and fundamental sub‐molecular mechanisms for the process of both ACSL6‐FLI1 and FLI1‐autophagy interactions. Collectively, in the current study we propose the ACSL6‐FLI1‐COL‐autophagy axis as a potential therapeutic target for improving LC radiotherapy.

## 5 Experimental Section

### Cell Lines and Culture Conditions

WT human LC cell lines, H460, A549, and H1299, were obtained from the Institute of Cell Biology, Chinese Academy of Sciences (Shanghai, China). These cell lines were confirmed using short tandem repeat analysis. H460 and H1299 cells were grown in RPMI‐1640 medium (Procell, PM150210, Wuhan, China), whereas A549 cells were cultured in DMEM (Procell, PM150110), both of which were supplemented with 10% fetal bovine serum (Gibco, 2 064 652, USA), penicillin (100 IU mL^−1^) and streptomycin (100 µg mL^−1^) and cultured at 37 °C under a humidified 5% CO_2_ atmosphere. The monoclonal ACSL6 KO H460 cell line was constructed via the CRISPR‐Cas9 technology combined with the blasticidin eukaryotic resistance screening (Blasticidin total lethal concentration, 3 µg mL^−1^; maintenance concentration, 1.5 µg mL^−1^). After two rounds of drug sieve (3 days/round), the screened cells were maintained in RPMI‐1640 medium supplemented with 10% FBS, 1% sodium pyruvate, and 1.5 µg mL^−1^ blasticidin.

### Vector Construction and Cell Transfection

The cells were transfected with siRNA oligonucleotides siFLI1 #1/2/3, siCOL2A1 #1/2/3, siCOL4A3 #1/2/3, and siCOL9A3 #1/2/3, and FLI1 negative control or overexpression plasmids (Genomeditech Co., Ltd., Shanghai, China). The siRNAs were mixed with Lipofectamine 2000 to form siRNA–lipidosome complexes. Single guide RNAs (sgRNAs) were designed using the CRISPR design software. Oligonucleotides corresponding to sgRNAs were synthesized and cloned into a lenti‐CRISPRv2 vector purchased from Genomeditech, and the lentiviral particles were packaged into 293T cells according to the manufacturer's protocol. The H460 cells were plated at a density of 1 × 10^5^ cells well^−1^ in 12‐well plates. After 24 h, the medium was replaced with 0.5 mL of lentiviruses and 0.5 mL of fresh medium containing 5 µg mL^−1^ Polybrene (Sigma–Aldrich, Catalog #H9268‐10G, Germany) and incubated for 24 h, which was followed by exchange with fresh medium and culture for another 48 h. The transfected cells were selected on 1 µg mL^−1^ puromycin for 1 week and the success of transfection was confirmed using agarose gel electrophoresis. Human sgRNA sequences were as follows:

**Table:** 

ACSL6 F	5′‐CTATGGGCACACAGACCATG‐3′
ACSL6 R	5′‐ GGAAATGAGGAAGCTCCAG‐3′
sgRNA sequencing primer	GAAGAAGCCCTGAAAGAGAG‐3′
siFLI1#1 F	5′‐ GUUCACUGCUGGCCUAUAA‐3′
siFLI1#1 R	5′‐ UUAUAGGCCAGCAGUGAAC‐3′
siFLI1#2 F	5′‐ CCCUUCUGACAUCUCCUACAU‐3′
siFLI1#2 R	5′‐ AUGUAGGAGAUGUCAGAAGGG‐3′
siFLI1#3 F	5′‐ CGUCAUGUUCUGGUUUGAGAU‐3′
siFLI1#3 R	5′‐ AUCUCAAACCAGAACAUGACG‐3′
siCOL2A1#1 F	5′‐ UGGCAAAGAUGGUGAGACA‐3′
siCOL2A1#1 R	5′‐ UGUCUCACCAUCUUUGCCA‐3′
siCOL2A1#2 F	5′‐ GCAAGGAGACAGAGGAGAA‐3′
siCOL2A1#2 R	5′‐ UUCUCCUCUGUCUCCUUGC‐3′
siCOL2A1#3 F	5′‐ GGGUGACGUUGGUGAGAAA‐3′
siCOL2A1#3 R	5′‐ UUUCUCACCAACGUCACCC‐3′
siCOL4A3#1 F	5′‐ CGGGUGAUAUGGGAAAGAA‐3′
siCOL4A3#1 R	5′‐ UUCUUUCCCAUAUCACCCG‐3′
siCOL4A3#2 F	5′‐ AGGCUUAAAUGGAUUGAAA‐3′
siCOL4A3#2 R	5′‐ UUUCAAUCCAUUUAAGCCU‐3′
siCOL4A3#3 F	5′‐ CCAGCAUACCCACACAAAU‐3′
siCOL4A3#3 R	5′‐ AUUUGUGUGGGUAUGCUGG‐3′
siCOL9A3#1 F	5′‐ CAGUUAGCCGCGCACCUAA‐3′
siCOL9A3#1 R	5′‐ UUAGGUGCGCGGCUAACUG‐3′
siCOL9A3#2 F	5′‐ GGCGCUACUGACCUUCAGU‐3′
siCOL9A3#2 R	5′‐ ACUGAAGGUCAGUAGCGCC‐3′
siCOL9A3#3 F	5′‐ GGACGGCAUUGACGGAGAA‐3′
siCOL9A3#3 R	5′‐ UUCUCCGUCAAUGCCGUCC‐3′

For the DLR assay, the WT COL2A1, COL4A3, and COL9A3 promoters were subcloned into pGL3 vectors to construct the PGL3‐basic‐H_COL2A1/COL4A3/COL9A3 Promoter (−2000 to + 50) WT.

### The CDX Model of LC

All of the animal research in this research complied with the 3Rs strategy for replacement, reduction, and refinement in the use of experimental animals, and had been approved by the Committee on Ethics of Medicine of Naval Medical University. Five‐ to six‐week‐old male nude BALB/c mice were purchased from the Shanghai Laboratory Animal Center of the Chinese Academy of Science. All living conditions and protocols were approved by the Naval Medical University Institutional Animal Care and Use Committee, in accordance with the Guide for Care and Use of Laboratory Animals published by the US NIH (publication No. 96‐01). After anesthetizing nude mice with isoflurane, H460 NC/KO cells (2 × 10^6^) were subcutaneously injected into the right flank of nude mice. Tumor size was monitored every three days using calipers. Specifically, tumor volume (mm^3^) was calculated using the following formula: *V = a × b × c/2*, where a, b, and c represent the length‐, width‐, and depth‐axis diameters, respectively. When the tumor size reached a volume of 100–150 mm^3^, the mice were assigned to the control and radiotherapy groups. A self‐developed local irradiation device was used for local irradiation of the mouse subcutaneous tumor, which was precisely delivered via a flexible tunable multi‐leaf collimator. When the tumor size reached 200 mm^3^, the mice were sacrificed and subcutaneous tumors were removed.

### The Primary LC Model

To improve the mouse lung tumor model, a Lox–Stop–Lox Kras conditional mouse strain (referred to as LSL‐Kras G12D) was used, in which the expression of oncogenic Kras is controlled by a removable transcriptional termination stop element. The endogenous Kras locus was targeted in the LSL‐Kras G12D strain; therefore, endogenous levels of oncogenic K‐Ras G12D protein were expressed following the removal of the stop element. The application of AdenoCre removed the stop element from the LSL‐Kras G12D allele, which allowed to control the timing, location, and multiplicity of tumor initiation. Conditional K‐Ras^G12D/+^ (LSL‐K‐Ras^G12D/+^) mice heterozygous for the G12D mutation were generated as previously reported (Figure S4a, Supporting Information). To reproduce K‐Ras^G12D/+^ mice, heterozygous K‐Ras^G12D/+^ mice were crossed with WT C57BL/6 mice, and the genotypes of K‐Ras^G12D/+^ were screened and retained using PCR and agarose gel electrophoresis. To induce non‐small cell lung cancer in mice, a lentivirus carrying sequences of both lung‐specific Cre adenovirus (Cyagen Biosciences, Suzhou, China) and mACSL6‐gRNA (Figure S5a, Supporting Information), which were instilled into the bronchi of the K‐Ras^G12D/+^ mice at a dosage of 5 × 10^5^ TU/mouse. These mice were divided into five groups as follows (five in each group, 10‐weeks‐old): Group A: WT; Group B: Tumor only; Group C: Tumor only + IR; Group D: Tumor only + gRNA *ACSL6*; Group E: Tumor only + gRNA *ACSL6* + IR. Subsequently, the mice were reared for 12 weeks under a specific pathogen‐free environment. In addition, the Cre‐tumor only + IR and gRNA‐*ACSL6*‐tumor + IR groups were subjected to local irradiation at the 8th week and the left lung of each mouse was irradiated. Before dissecting the lungs, the lung function was assessed. Finally, the lung tissue was harvested for the follow‐up tissue analysis (the left lung was fixed and the right lung was cryopreserved). Mouse gRNA (LV‐U6>{mAcsl6‐gRNA‐A1}‐U6>{mAcsl6‐gRNA‐A2}‐EFS>hCas9/P2A/Cre) sequences were as follows:

gRNA #A1:GCAGTAGATGGCCCGGTGCA‐GGG
gRNA #A2:AGAGTACAAGGCCTGATACC‐AGG

### Ionizing Radiation and Local Irradiation

All irradiation operations were performed at the Radiation Center of the Naval Medical University. For whole‐body exposure, samples were directly irradiated at various doses using a ^60^Co gamma source at room temperature. For local radiation, a self‐made multi‐leaf collimator system was used to ensure delivery accuracy. In brief, ultrathin lead blocks, which were polished smoothly and shaped into a special “L” profile, were put together firmly on the targeted irradiated area of mice chest. To ensure the accuracy of the irradiated area, the targeted area was confirmed every time before irradiation using a laser beam perpendicular to the mouse skin through the reserved channels for gamma rays, which may be adjusted slightly depending on the feedback of the calibration procedure.

### RT‐qPCR

Total RNA was extracted from cells using the Trizol method (QIAGEN, Germany) and reverse transcribed into complementary DNA (cDNA) using a cDNA Synthesis Kit (Takara, RR036A, Japan), according to the manufacturer's instructions. RT‐qPCR was performed using a SYBR Green PCR Kit (Takara, RR042A, Japan) on a 7500 Fast Real‐Time PCR machine (Applied Biosystems/Thermo Fisher Scientific). The following primers were used:

**Table:** 

ACSL6 forward	5′‐ GCACGGCGATCTGTGATTG‐3′
ACSL6 reverse	5′‐ GGCGGAACACCTGGTACAT‐3′
FGF2 forward	5′‐ AGAAGAGCGACCCTCACATCA‐3′
FGF2 reverse	5′‐ CGGTTAGCACACACTCCTTTG‐3′
LAMB3 forward	5′‐ GCAGCCTCACAACTACTACAG‐3′
LAMB3 reverse	5′‐ CCAGGTCTTACCGAAGTCTGA‐3′
PPP2R2C forward	5′‐ CCGGGTCGTCATCTTCCAG‐3′
PPP2R2C reverse	5′‐ GTTGGTGGACAGGAGTGAGT‐3′
COL9A3 forward	5′‐ GTGGATGGTCTGACTGGACG‐3′
COL9A3 reverse	5′‐ GGGCAGATACTTGGGCACTG‐3′
COL4A3 forward	5′‐ AGCAAGGGTTGTGTCTGTAAAG‐3′
COL4A3 reverse	5′‐ CAGAAAATCCTGGCAATCCACT‐3′
COL2A1 forward	5′‐TGGACGATCAGGCGAAACC‐3′
COL2A1 reverse	5′‐ GCTGCGGATGCTCTCAATCT‐3′
COL4A4forward	5′‐ GCTGCGGATGCTCTCAATCT‐3′
COL4A4reverse	5′‐ ACCTTTAACGGCACCTAAAATGA‐3′
NTRK1 forward	5′‐ AACCTCACCATCGTGAAGAGT‐3′
NTRK1reverse	5′‐ TGAAGGAGAGATTCAGGCGAC‐3′
NRIP1 forward	5′‐ GGATCAGGTACTGCCGTTGAC‐3′
NRIP1 reverse	5′‐ CTGGACCATTACTTTGACAGGTG‐3′
TFAP2C forward	5′‐ CTGTTGCTGCACGATCAGACA‐3′
TFAP2C reverse	5′‐ CTCAGTGGGGTTCATTACGGC‐3′
FOXA1 forward	5′‐ GCAATACTCGCCTTACGGCT‐3′
FOXA1reverse	5′‐ TACACACCTTGGTAGTACGCC‐3′
FLI1 forward	5′‐ CAGCCCCACAAGATCAACCC‐3′
FLI1 reverse	5′‐ CACCGGAGACTCCCTGGAT‐3′
MYC forward	5′‐ GGCTCCTGGCAAAAGGTCA‐3′
MYC reverse	5′‐ CTGCGTAGTTGTGCTGATGT‐3′
GAPDH forward	5′‐ TGTGGGCATCAATGGATTTGG‐3′
GAPDH reverse	5′‐ ACACCATGTATTCCGGGTCAAT‐3′

### Western Blotting

Cells were lysed using RIPA lysis buffer to extract total protein, which was then separated by SDS‐PAGE and transferred onto PVDF membranes. After blocking with 5% milk for 1 h at room temperature, the membranes were incubated, with shaking, overnight with primary antibodies at 4 °C, followed by incubation with secondary antibodies at room temperature for 1 h. The following primary antibodies were used in this study: anti‐β‐actin (ABclonal, AC004, Wuhan, China), anti‐FLI1 (ABclonal, A5644, Wuhan, China), anti‐COL2A1 (Signalway Antibody, 38 254, USA), anti‐COL4A3 (Signalway Antibody, 54 194, USA), anti‐ATG12‐5 (CST, 2011S, USA), anti‐LC3II/LC3I (CST, 4108, USA), anti‐Bcl‐2 (CST, 4223S, USA), anti‐Bax (CST, 41 162, USA), anti‐c‐Caspase3 (CST, 9662, USA), anti‐P53 (CST, 2527T, USA), anti‐p‐P53 (CST, 82 530, USA), and anti‐P21 (CST, 2947T, USA), anti‐P62 (proteintech, 18420‐1‐AP, Wuhan, China), anti‐ACSL6 (Aifang biological, 03 893, Hunan, China)

### Chemical Inhibitors

3‐MA, ETO, and CPT were purchased from MedChemExpress (USA) and stored at 4 °C (protected from light). Culture medium was used to dissolve 3‐MA and dimethyl sulfoxide (DMSO) was used as a medium to dissolve ETO and CPT (DMSO <0.1% in all experiments). H460 cells were treated with 2.5 mm 3‐MA, 0/250/500 µm ETO, and 0/0.25/0.5/1 µm CPT, respectively, by incubating at 37 °C for 24 h. The cells were lysed using Protein Lysis Buffer and ultrasonication, and the soluble fraction obtained after centrifugation was used for western blot analysis.

### Cell Viability Assay

Cell viability was assessed using the CCK8 assay. H460 cells were divided into three groups: negative control, knockout, and overexpression. Cells in the logarithmic growth phase were seeded evenly onto 96‐well plates (3000 cells/well) 12 h before irradiation (8 Gy, 1 Gy min^−1^). At 24, 48, and 72 h of irradiation, 10 µL of the CCK‐8 solution was added to each well and the plates were incubated in an incubator for another 1–2 h. The optical density (OD) at 450 nm was measured using a microplate reader (BioTek, NEO2SM, USA), and the cell viability rate was calculated using the following equation:

(1) $$\mathrm{Cell}\;\mathrm{Viability}=\frac{\left(\mathrm{Processing}\;\mathrm{Group}\;\mathrm{OD}\;\mathrm{Value}\right)-\left(\mathrm{Blank}\;\mathrm{Group}\;\mathrm{OD}\;\mathrm{Value}\right)}{\left(\mathrm{Negative}\;\mathrm{Control}\;\mathrm{OD}\;\mathrm{value}\right)-\left(\mathrm{Negative}\;\mathrm{Blank}\;\mathrm{Group}\;\mathrm{OD}\;\mathrm{value}\right)}$$

### Clonogenic Survival Assay

Radiosensitivity of different cell lines was determined using a clonogenic survival assay. Briefly, exponentially growing cells at 70–80% confluence were trypsinized and replated at various densities in 6‐well plates (200, 400, 800, 1600, 3200 cells well^−1^). After 12 h of incubation, the adhered cells were exposed to a series of IR doses (0, 2, 4, 6, and 8 Gy). After two weeks of incubation, the colonies were fixed with ethanol and stained with 0.05% crystal violet diluted in PBS. The plates were then imaged and counted automatically using a Colony Imaging and Analysis System (ScanMarker i800plus, MICROTEK, China), and only colonies consisting of >50 cells were counted. To analyze radiobiological parameters, such as the surviving fraction (SF), plating efficiency (PE), and surviving fraction at 2 Gy (SF2), the following formulas were adopted:

(2) $$\begin{array}{ccc} \mathrm{SF} & = & \left(\mathrm{Mean}\;\mathrm{Colony}\;\mathrm{Count}\right)/\left[\left(\mathrm{Cells}\;\mathrm{Inoculated}\;\mathrm{for}\;\mathrm{Irradiation}\right)\right.\\ & & \left.\times \;\left(\mathrm{Plating}\;\mathrm{Efficiency}\right)\right] \end{array}$$

(3) $$\mathrm{PE}=\left(\mathrm{Mean}\;\mathrm{Colony}\;\mathrm{Count}\right)/\left(\mathrm{Cells}\;\mathrm{Inoculated}\;\mathrm{for}\;\mathrm{Control}\right)$$

Finally, the mean value and standard deviation of the SFs were calculated, and the GraphPad Prism 9 software was used to fit the survival curve y = exp(−αx − βx^2^) for comparison between the groups using the LQ model.

### Immunofluorescence

Cells were washed with PBS twice, fixed with 4% paraformaldehyde, and permeabilized with 0.5% Triton X‐100 (1 mL well^−1^) for 20 min on ice. Subsequently, the coverslips were blocked with 5% goat serum for 1 h at room temperature. Thereafter, the coverslips were incubated overnight at 4 °C in primary antibody, anti‐γH2AX (ABclonal, AP009, Wuhan, China), anti‐ACSL6 (Aifang biological, AF03893, Hunan, China), anti‐FLI1 (Boster Biological Technology, Wuhan, China. Catalog#A00399), and then in secondary antibody (Alexa Fluor 488‐conjugated; JACKSON 111‐167‐003) for 1–1.5 h; this was followed by incubation in DAPI solution for another 10 min. Finally, images were acquired with a Zeiss microscope (Axio Vert.A1; Carl Zeiss, German) using a 63× oil immersion lens before processing using ImageJ and Cell Profiler.

### Apoptosis

Apoptotic cell death was detected using flow cytometry. Cells were seeded in six‐well plates and treated with or without 8 Gy IR, followed by harvesting and washing twice with cold PBS after 48 h. The pellets were resuspended in 100 µL of 1× binding buffer and stained with Annexin‐V‐FITC (5 µL) and PI staining solution (5 µL) for 15 min. Finally, 400 µL of 1× binding buffer was added to each well and the samples were detected using a flow cytometer (Cytoflex, BECKMAN COULTER Life Sciences, USA) within 1 h.

### ROS Analysis

Cells were detected using flow cytometry or laser confocal microscopy. DCFH‐DA was diluted with serum‐free culture medium at 1:1000 to achieve a final concentration of 10 µmol L^−1^. The cells were collected and suspended in diluted DCFH‐DA at a concentration of 1 million to 20 million cells mL^−1^, and then incubated for 20 min in a cell culture incubator set at 37 °C. The mixture was blended and inverted every 3–5 min to bring the probe and cells into contact. The cells were washed three times with serum‐free cell culture medium to ensure complete removal of DCFH‐DA. The fluorescence intensity before and after stimulation was measured at an excitation wavelength of 488 nm and an emission wavelength of 525 nm. Additionally, the fluorescence intensity under the GFP channel was visualized using fluorescence microscopy.

### Cell Cycle Analysis

A total of 5 × 10^5^ cells were harvested, washed twice with PBS, and then fixed in cold ethanol (70%). The cells (at least 1 × 10^4^ cells) were stained with PI (20 µg mL^−1^) and RNase A (0.2 mg mL^−1^) for 30 min before being analyzed using flow cytometry. The data were analyzed using the FlowJo software. All cell cycle analyses were performed in triplicate and repeated at least three times.

### SA‐β‐gal Assay

Cells were washed three times with PBS and fixed in stationary liquid for 15 min at room temperature. Subsequently, 1 mL cell senescence β‐galactosidase staining solution was added to each well, followed by sealing of the 6‐well plate with Parafilm and incubating it overnight in a CO_2_‐free cell culture incubator at 37 °C until the cells were stained blue. Finally, the cells were observed under a light microscope, and percentage of the total number of counted cells in each well was regarded as the proportion of the SA‐β‐gal activity‐positive cells. The results were expressed as mean of triplicate values ±SD.

### Immunohistochemistry

Briefly, sections were dewaxed and rehydrated. Antigen retrieval was performed by pretreating the slides with citrate buffer (pH 6.0) in a microwave oven for 23 min. The slides were then incubated with PBS containing 3% hydrogen peroxide for 25 min, followed by PBS containing 5% BSA for 30 min before overnight incubation at 4 °C with the primary antibody. After slight shaking and drying, the tissues were covered with a secondary antibody and incubated at room temperature for 50 min. Subsequently, reactions with diaminobenzidine and counterstaining with Mayer's hematoxylin were performed. At least eight fields were randomly selected to score each slide. The proportion of DAB chromogenic‐positive cells was calculated using ImageJ, with the results reported as integrated optical density (IOD) and area.

### HE Staining

The harvested lung tissue was isolated, fixed in 4% paraformaldehyde, embedded in paraffin, and sliced into 4 µm thick sections. After deparaffinization and rehydration, the sections were stained with HE, according to standard protocols. Observations were made under a light microscope after sections were dehydrated and cleared with xylene.

### Acetyl‐CoA Assay

The procedures were conducted in accordance with the manufacturer's instructions (Solarbio, Acetyl‐CoA Detection Kit, BC0980, China). Briefly, 5 × 10^6^ cells were collected and added to a mixture of 0.99 mL extract I and 0.01 mL extract II. After ultrasonication on ice, the cells were centrifuged at 8000 × *g* for 10 min at 4 °C, and the absorbance at 340 nm of the supernatant was detected using an ultraviolet spectrophotometer (Biotek Synergy Neo2, Agilent, USA).

### Glucose Assay

Glucose levels were detected according to the manufacturer's instructions (Solarbio, Glucose Detection Kit, BC2505, China). First, cells were seeded in a 10 mm dish prior to detection. Next, 5 × 10^6^ cells were resuspended in 1 mL of distilled water and subjected to ultrasonic cell disruption on an ice‐bath. The samples were then boiled in a metal bath for 10 min after which they were centrifuged at 8000 × *g* for 10 min at 25 °C and the supernatant was collected. The absorbance of the samples was measured at 505 nm using a microplate reader.

### Lactate Assay

The lactate assay was used to detect changes in the levels of lactate in the different groups, following the protocol prescribed by the manufacturer (Solarbio, Lactate detection kit, BC2230, China). One milliliter of extract I was added to 5 × 10^6^ exponentially growing cells in a 10 mm dish. After ultrasonic cell lysis on ice, the samples were centrifuged at 13 680 × *g* for 10 min at 4 °C. A mixture of 0.8 mL of the supernatant and 0.15 mL of extract II was prepared and centrifuged under the same conditions. The supernatant was collected for subsequent testing. Finally, the optical density at 570 nm was measured using a multimode reader (BioTek, USA).

### Co‐Immunoprecipitation (Co‐IP)

Protein lysate (450 µL) containing 1% each of protease and phosphatase inhibitors was added onto cells cultured in a culture dish (6 mm), and the mixture was then transferred to a 1.5 mL tube and centrifuged at 13 680 × *g* for 15 min at 4 °C. Next, 80 µL of the supernatant was blended with the 5× protein loading buffer, with the supernatant used as the input group. The remaining supernatant was incubated overnight with 2 µg of anti‐ACSL6 (Aifang biological, AF03893, Hunan, China) at 4 °C for immunoprecipitation. Resuspended beads (Beyotime, BeyoMag Protein A+G beads, P2108, China) were rinsed twice with 300 µL of the NETN‐100 reagent on a magnetic stand. The magnetic beads were added into 1.5 mL tubes that had been incubated overnight and left to incubate for 2–3 h at 4 °C. After rinsing twice with 300 µL NETN‐300 and IP lysis buffer, respectively, the beads were sequentially resuspended in IP lysis and 5× protein loading buffer and boiled for 7 min. The subsequent steps were performed as described for western blotting.

### Dual‐Luciferase Reporter Assay

293T cells were cultured in 96‐well plates for 24 h and transfected with plasmids for another 24 h (control group: FLI1 NC, COLs WT, and pRL‐TK plasmids; overexpression group: FLI1 OE, COLs WT, and pRL‐TK plasmids). The cell culture medium was discarded and the cells were washed twice with PBS. Then, add 100 µL of the recommended amount of 1× cell lysis buffer to each well in a 96‐well plate. The mixture was then incubated or shaken for 5 min at 4 °C, and mixed by pipetting up and down. The cell lysate was transferred to a 1.5 mL centrifuge tube and centrifuged at 17 320 × *g* for 1 min at 4 °C. The supernatant was collected and used for experiments. The cell lysate (20 µL) was taken in a 96‐well plate, and 100 µL of the luciferase substrate (preequilibrated to room temperature) was added to the plate. The activity of the firefly luciferase reporter gene was detected rapidly using a microplate reader. Eventually, 100 µL of freshly prepared Renilla substrate solution was added to the above reaction solution and mixed rapidly. The Renilla luciferase reporter gene activity was detected immediately.

### Lung Function Test in Mice

All mice were anesthetized and tracheostomized, and lung function was assessed using an eSpira Forced Maneuvers System (EMMS; Edinburgh, UK). The supporting Anires2005 analysis software was used to analyze pulmonary functional parameters, such as pressure (P), volume (V), flow (F), respiratory dynamic compliance (Cdyn), forced vital capacity (FVC), and FEV (1)/FVC% (FEV1: Forced expiratory volume within 1 s).

### Detection of Autophagic Flux

The PGMLV‐CMV‐RFP‐GFP‐hLC3‐Puro lentivirus was used to generate H460‐RFP‐GFP‐hLC3 cells, and the fluorescence intensity of GFP^+^ and RFP^+^ autophagosomes and GFP^−^ and RFP^+^ autolysosomes were determined using ZEISS microscope.

### Micro‐CT

GEMM of different groups were subjected to anesthetization and the in vivo imaging examination using a Micro‐CT (In Vivo CT 80, SCANCO MEDICAL, Swiss) to determine the tumor volumes. Next, comparisons of the calculated tumor volume between groups were performed and the responses of the tumors to different interventions were assessed in high fidelity.

### Bioinformatic Assays

Total RNA was extracted from BEAS 2B and H460 cells in both the NC and ACSL6‐KO groups. RNA sequencing was performed to identify DEGs between the groups. Differential expression analysis between two groups was performed using the DESeq R package, with *p*‐values adjusted using the Benjamini–Hochberg method to control the false discovery rate (FDR). The cutoff value for DEGs was set as |log2[fold change (FC)] | ≥1 and FDR ≤ 0.05. The KEGG enrichment analysis was employed to investigate the biological functions of DEGs using the DAVID database (https://david.ncifcrf.gov/). Statistical significance was set at *p* < 0.05.

### Human Lung Tumor Sampling

This subject about the human research participants had been approved by the Committee on Ethics of Medicine of Naval Medical University. Before surgery, the patient consent statements regarding the devotion of the removed lung cancer tissue were assigned. The inclusion criteria for lung cancer tumors is as follows: tumors with a diameter greater than or equal to 3 cm, and T1 tumors were excluded. The sampled lung cancer tissue was guaranteed to be at least 0.5 cm in diameter.

### Statistical Analysis

Data are shown as mean ± SEM from at least three independent biological replicates of experiments unless otherwise specified. For the RT‐qPCR and CCK8 assay, the control group was used as the denominator for data normalization. The student's *t*‐test was used for comparison of two groups, whereas ANOVA was used for comparison of multiple groups. Generally, the GraphPad Prism 9 software was adopted for statistical comparisons, while other special software such as FlowJo, Anires2005 was used for the experimental data analysis. Results with *p* value <0.05 were considered statistically significant.

## Conflict of Interest

The authors declare no conflict of interest.

## Author Contributions

W.D., S.B., and Q.Z. contributed equally to this study. J.G. discovered the role of ACSL6 in regulating the LCRS, devised the concept, and supervised the project. J.G. and Q.Z. conducted biological experiments using the LLC cell line. W.D., S.B., Q.Z., W.H., Y.X., and X.L. performed in vitro and in vivo biological experiments. J.G. and W.H. designed and performed both transcriptomics and metabolomics experiments, while W.D., W.H., and K.F. conducted bioinformatics analysis. J.G. and W.D. designed the specific GEMM generation strategy, while W.D., X.X., Z.Z., X.C, and L.Y. raised, bred, and identified the mouse genotypes. H.J. conducted clinical sampling and performed the analysis. J.G., W.D., and Q.Z. wrote the manuscript, and J.G., Q.Z., and Q.Z. provided considerable editorial input. All the authors commented on this manuscript and approved its submission.

## Supporting information

Supporting Information

## Data Availability

The data that support the findings of this study are available from the corresponding author upon reasonable request.
