# Supplementary material for: Blocking ACSL6 Compromises Autophagy via FLI1‐Mediated Downregulation of COLs to Radiosensitize Lung Cancer
Source: Adv Sci (Weinh). 2024 Aug 29;11(40):2403202. doi: 10.1002/advs.202403202 (PMC11516120; doi:10.1002/advs.202403202)
Supplement: Supplementary file 1 — Supporting Information [file ADVS-11-2403202-s001.docx]

**SUPPLEMENTARY INFORMATION**

Blocking ACSL6 Compromises Autophagy via FLI1-Mediated Downregulation of COLs to Radiosensitize Lung Cancer

Wen Ding et al.


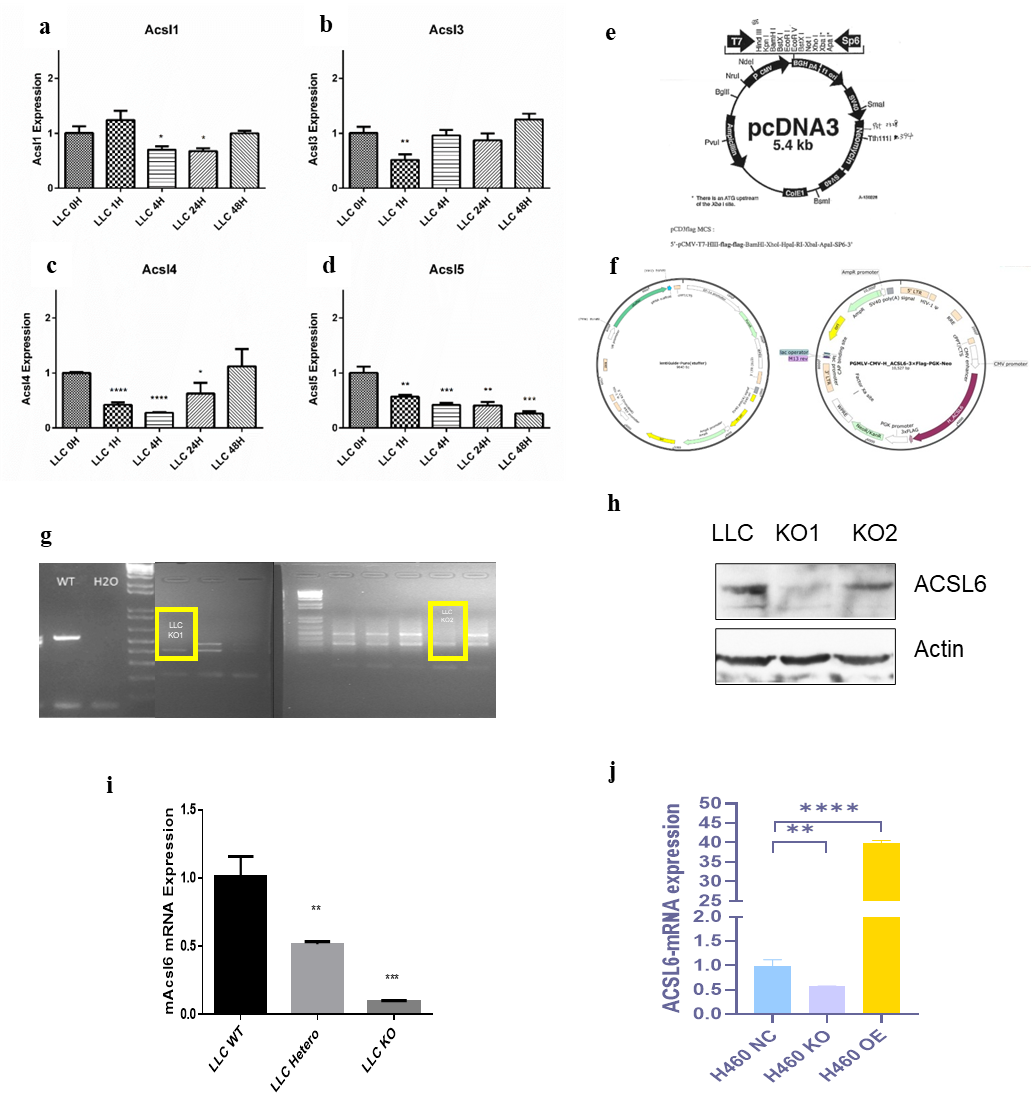


**Figure S1.** **Generation and confirmation of the ACSL6 differentially expressing-lung cancer cell lines.** At first, we determined the expression profile of all the Acsls in a murine lung cancer cell line (Lewis lung carcinoma, LLC) by qRT-PCR. After constructing the appropriate plasmids, the transfection was performed and the stable cell lines were obtained by repeating screening, confirmed by PCR products bands, mRNA expression, and the protein blotting bands. **a-d** RT**-**qPCR was performed to measure the expression of ACSL1, 3, 4, 5 in LLC at the indicated time points post IR. **e** Schematic diagram of the design for ACSL6 KO plasmid construction. **f** Schematic diagram of the design for ACSL6 OE plasmid construction. **g** Screening for the CRISPR CAS9 technique-aided ACSL6 KO individuals by PCR amplification bands. **h** WB analysis examined the protein level of ACSL6 in LLC ACSL6 WT/KO1/KO2 cell lines. **i, j** RT-qPCR was performed to measure the ACSL6 expression in various LLC and H460 cell lines.


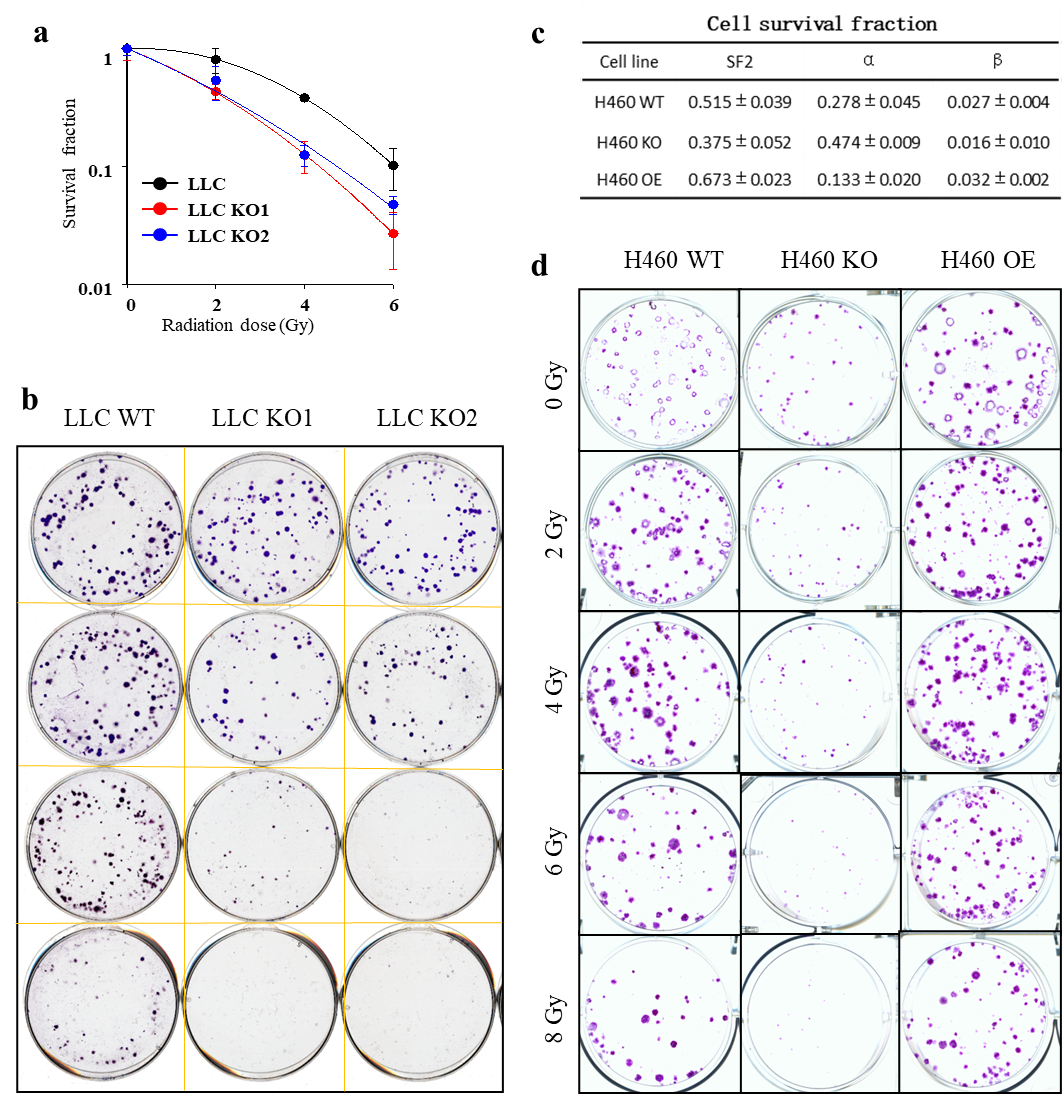


**Figure S2.** **The clonogenic assay using LLC and H460 cell lines to examine the radiosensitivity alterations by ACSL6 modulation. a** The absence of ACSL6 decreased the clonogenic ability of LLC according to the clonogenic assay after different doses of IR delivery. **b, d** Representative wells of the clonogenic assay with LLC and H460 exposed to a series doses of IR. **c** The calculated radiological parameters according to the clonogenic data of H460 cells. SF2 means the surviving fraction at 2Gy. The α and β mean the linear and the quadratic coefficient of Linear Quadratic Equation [SF=exp(-αx-βx^2^)], respectively.


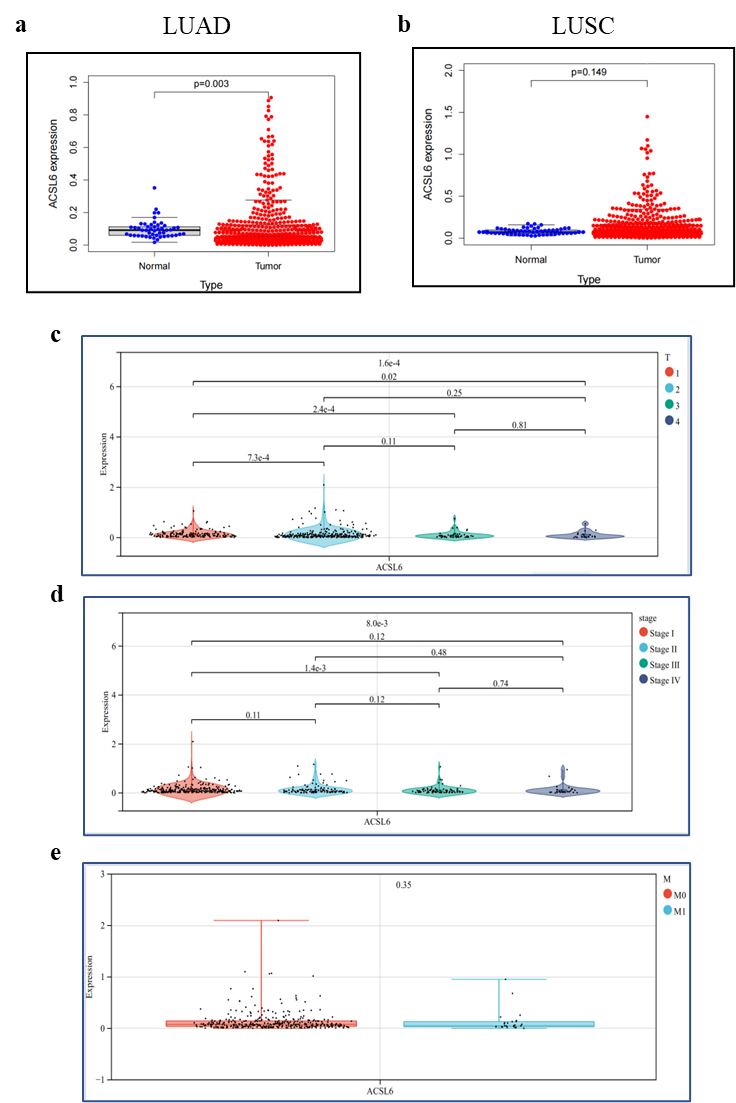


**Figure S3. Clinical investigation of the mRNA expression of ACSL6 in lung cancer using TCGA database. a, b** Differential expression analysis for ACSL6 between tumor and normal tissues referring to TCGA database for both LUAD and LUSC. **c, d, e** The expression analysis of ACSL6 of LUAD patients belonging to different T (e), Stage (f), and M (g) clinical grades.


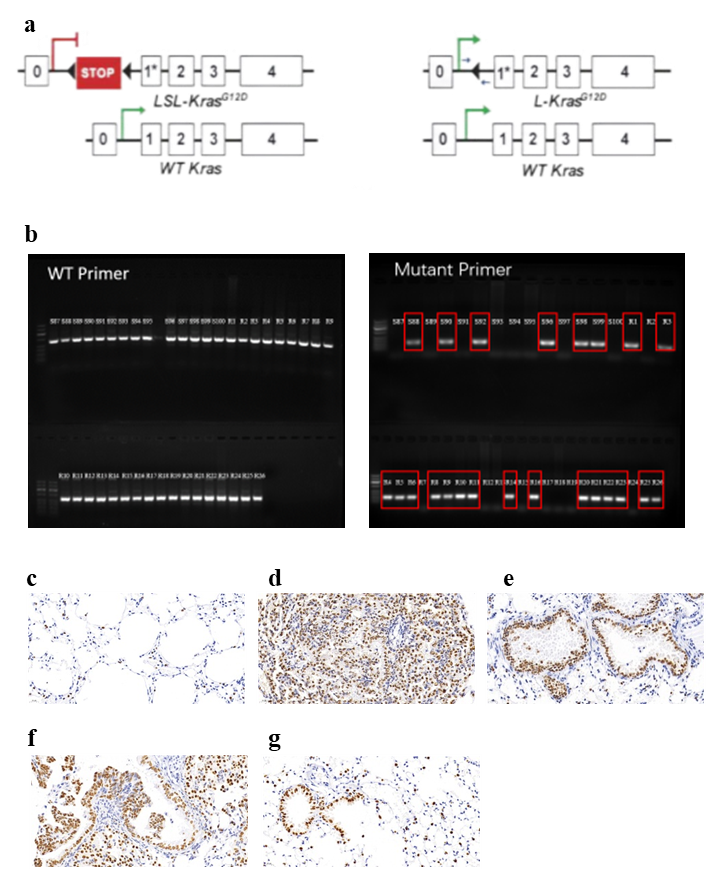


**Figure S4. a** The pattern diagram of the generation of the Kras^G12D^ mutated strains. **b** Agarose gel electrophoresis for Kras^G12D/+^ genotype. **c-g** IHC examination of tissues from Kras^G12D/+^ mice. Tissue sections were stained using anti-TTF-1 (j-n) antibodies (1×, 200× and 400× magnification). Panels **a** to **g** represent tissue sections from groups A to E, respectively.


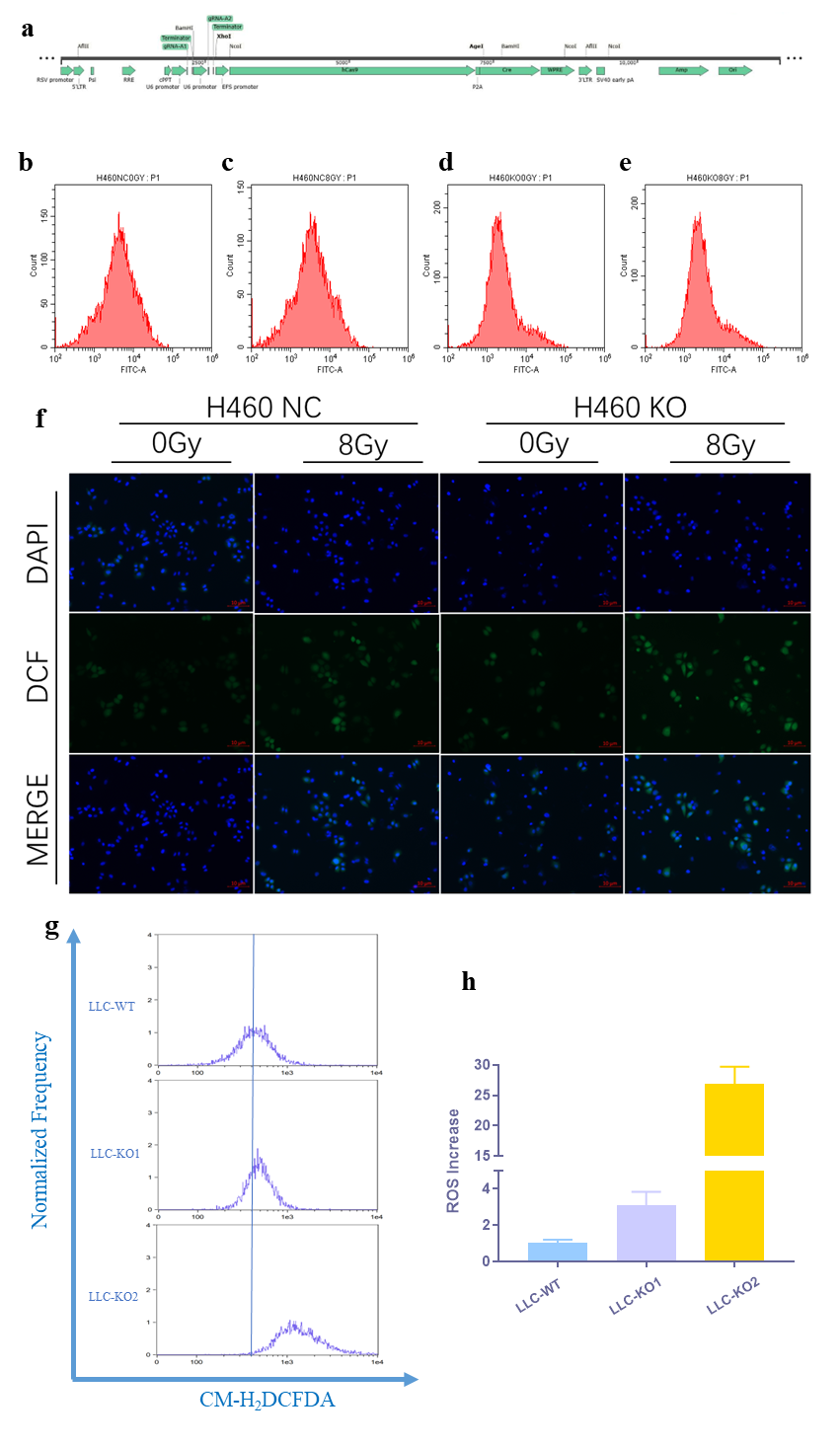


**Figure S5.** **Design of the CRISPR Cas-ACSL6 gRNA lentivirus for generation of GEMM and the evaluation of ROS changes in H460 cells. a** The sequence of CRISPR Cas-ACSL6 gRNA lentivirus. **b-e** Representative images of ROS detection in H460 NC/KO via flow cytometry after sham/IR. **f** Representative IF images of DCF detection for evaluating the ROS status in H460 NC/KO. **g** Representative images of the normalized frequency for ROS assessment in LLC WT/KO using FCM. **h** Quantification of the CM-H2DCFDA intensity for LLC cell lines to exhibit the ROS increase based on FCM detection.


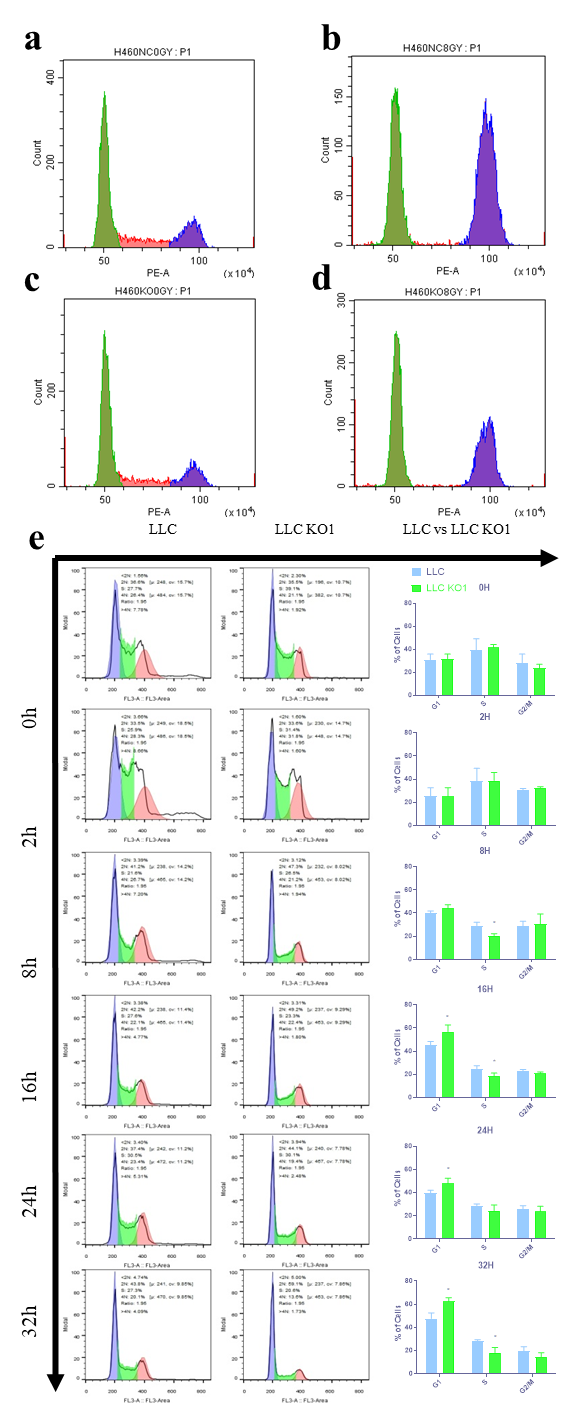


**Figure S6. The results of cell cycle in H460 and LLC cell lines at different timepoints after IR.** **a-d** The flow cytometry was conducted to detect the PI staining signals to measure the cell cycle of H460 ACSL6 NC/KO after sham/IR. **e** Representative histograms of the cell cycle analysis and the according quantitative bar graphs which reflect the percentage of G1, S and G2/M phase for LLC WT/KO at the indicated times points after IR.


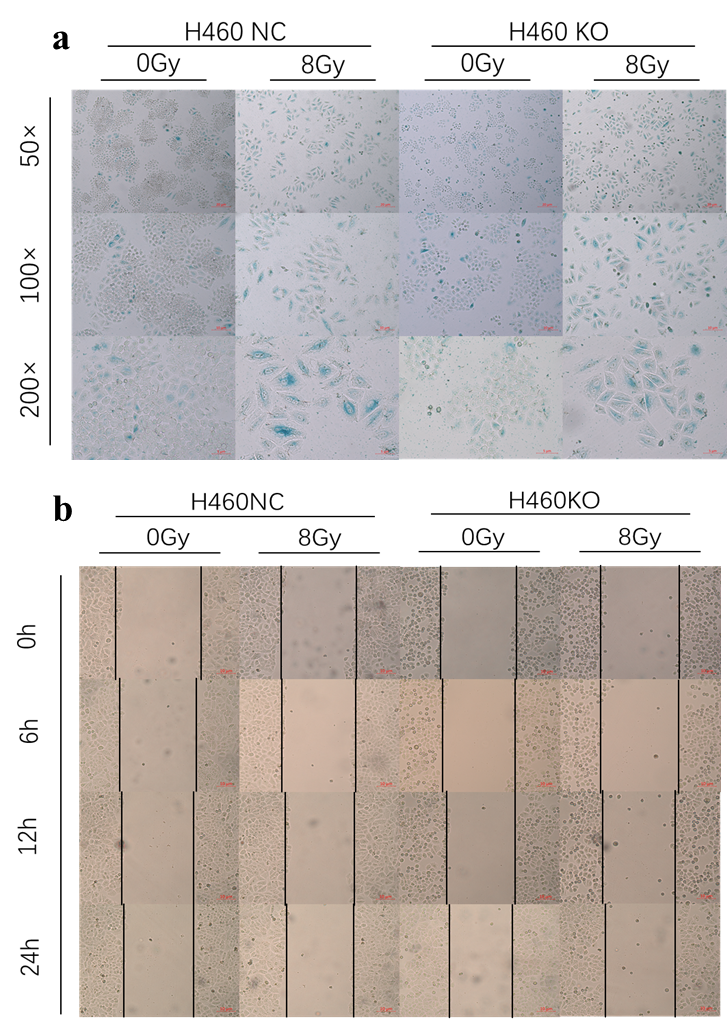


**Figure S7. Senescence and wound-healing determination using H460 cells. a** Representative images of senescence detection for H460 ACSL6 NC/KO by SA-β-gal assay. **b** Representative images of the wound-healing assay for H460 NC/KO after sham/IR.


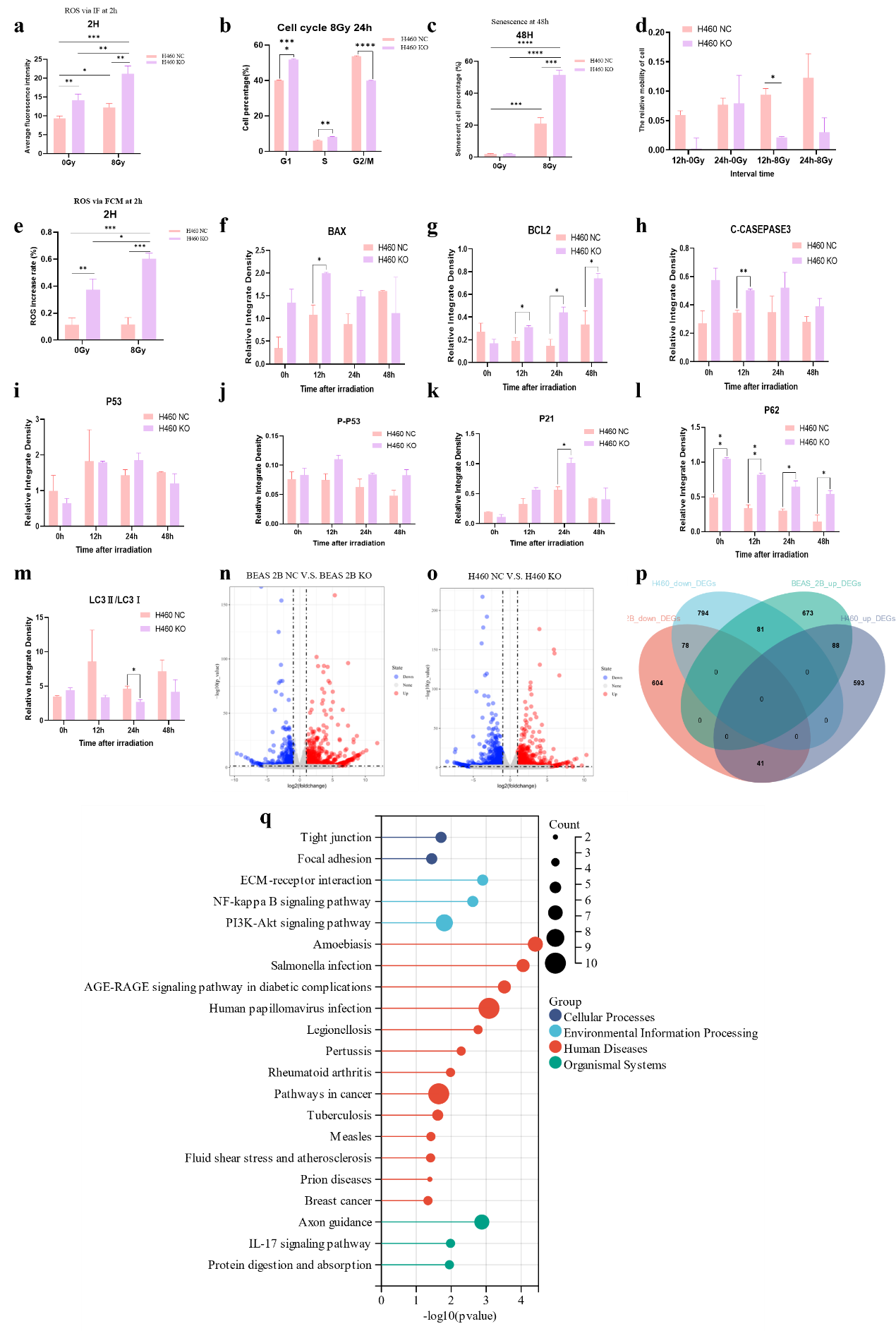


**Figure S8. a** The average fluorescence intensity of ROS is shown using a bar graph quantifying IF signals in H460 NC/KO cell lines at 2h post IR. **b** The H460 NC/KO cell percentages of G1, S and G2/M phases are examined by FCM. **c** The percentages of senescence population for each treatment group are shown here using a bar graph by SA-β-gal assay. **d** The cellular migration capability were assessed using the wound-healing assay. **e** The ROS level was investigated again via FCM in H460 NC/KO at 2h post IR. **f-m** Bar graphs show the relative integrate density of the corresponding WB bands in figure 3g. **n, o** The volcano maps show the identification of differentially expressed genes in BEAS 2B and H460 cells. **p** The Venn diagram shows the number of differential genes among the indicated treatment groups. **q** According to the transcriptome data mining, the bubble plot exposed the enriched pathways such as hsa04151: PI3K-Akt signaling.


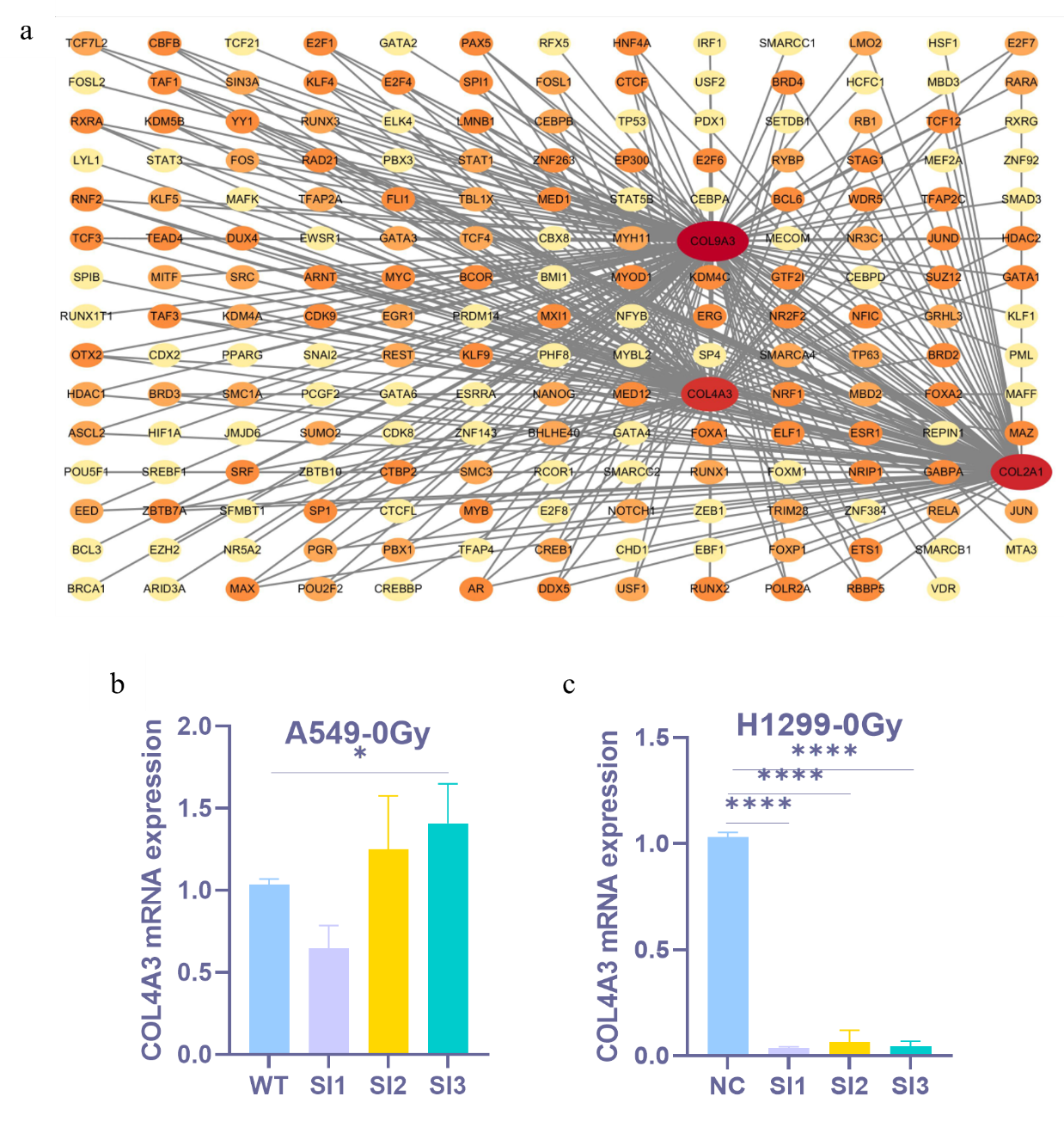


**Figure S9. a** The potential TFs of COL2A1, COL4A3, and COL9A3 were predicted via analyzing the reliable curated ChIP-Seq datasets in hTFtarget. **b, c** Determination of the COL4A3 expression by RT-qPCR after knocking down FLI1 in A549 **(b)** and H1299 **(c)** cells.


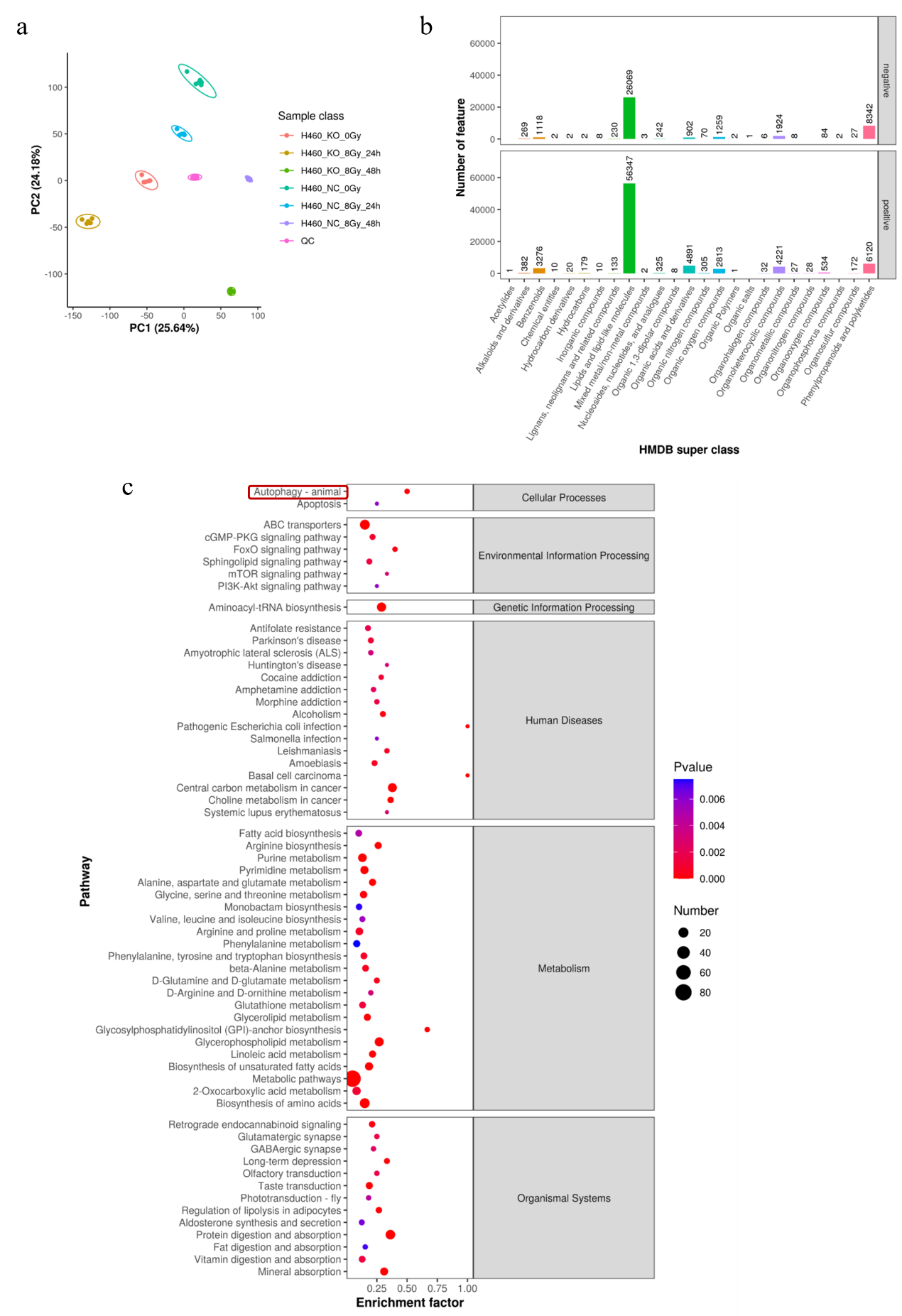


**Figure S10. Metabolomics examination and the bioinformatic survey including the super class clustering and KEGG analysis suggest autophagy as the terminal cellular processes. a** Principal Component Analysis (PCA) evaluation of H460 KO/NC cells after IR. **b** The super class clustering analysis referring to the HMDB exhibits the number of metabolites in different metabolic pathway. **c** The KEGG enrichment analysis of different metabolites indicates that autophagy is dramatically adjusted with strong enrichment factor (framed in a red rectangle).


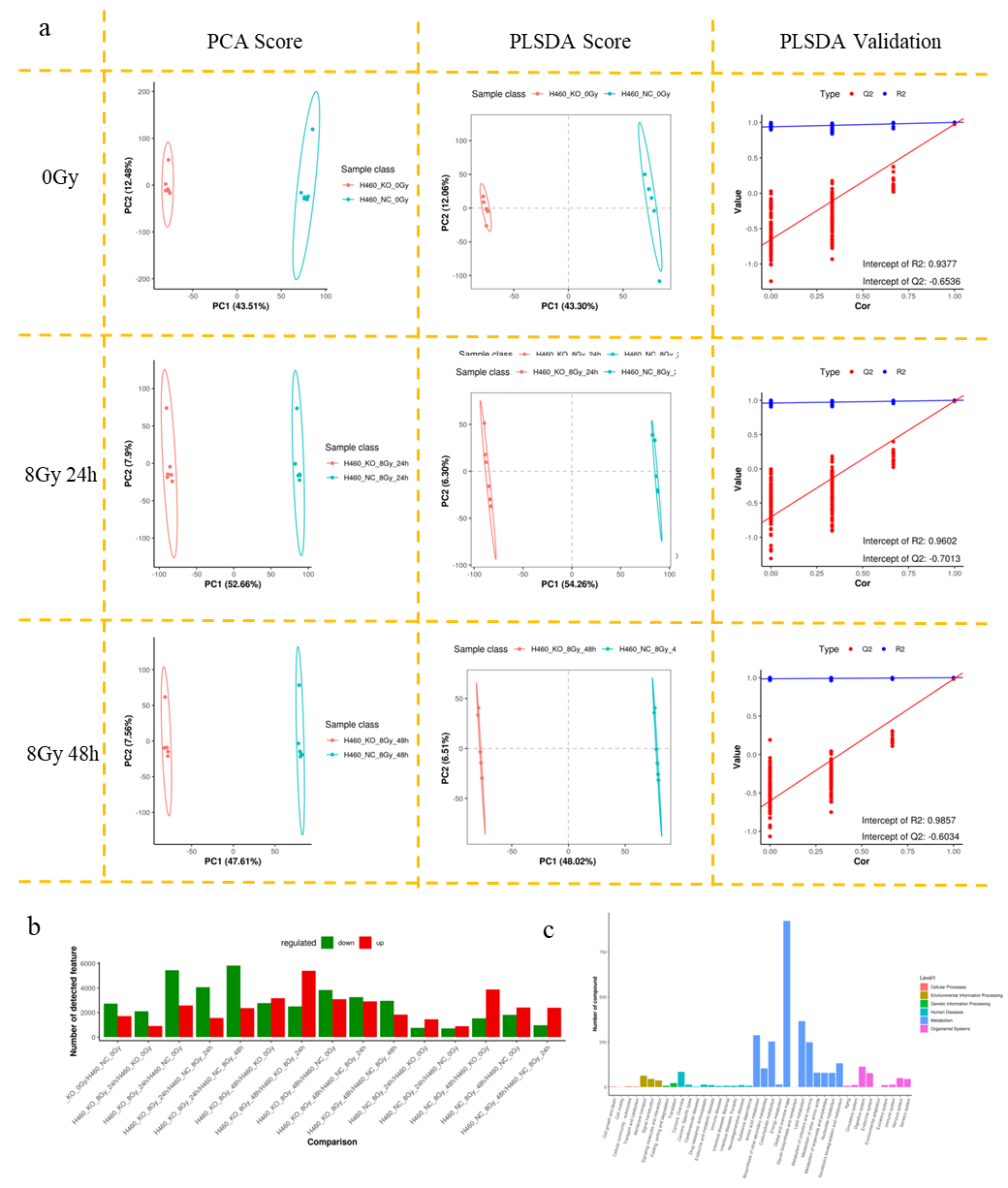


**Figure S11. Quality control of the metabolomics examination by PCA, PLS-DA and OPLS-DA analysis in H460 cells and the identification of the regulated compounds. a** PCA, PLS-DA, and OPLS-DA analysis were performed to ensure the quality control of the entire metabolomics analysis. **b** A summary of upregulated and downregulated metabolites in each indicated treatment group. **c** Identification of the number of detectable metabolites in different metabolic pathways.


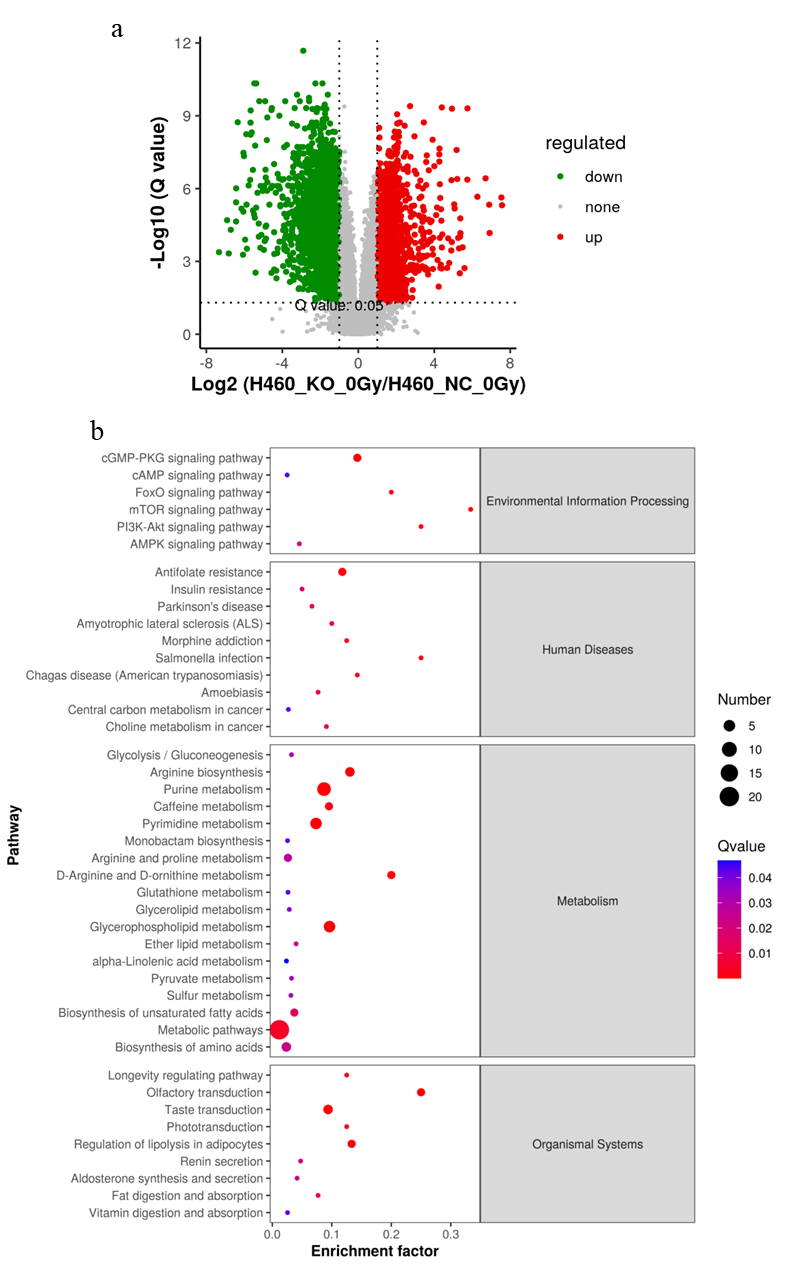


**Figure S12.** **Clustering and KEGG enrichment analysis of H460 NC/KO receiving no IR based on the metabolomic source data.** **a** The volcano map shows the identification of differentially expressed genes in H460 NC/ KO cells without IR. **b** KEGG enrichment analysis of differential metabolites in H460 NC/KO cells without IR.


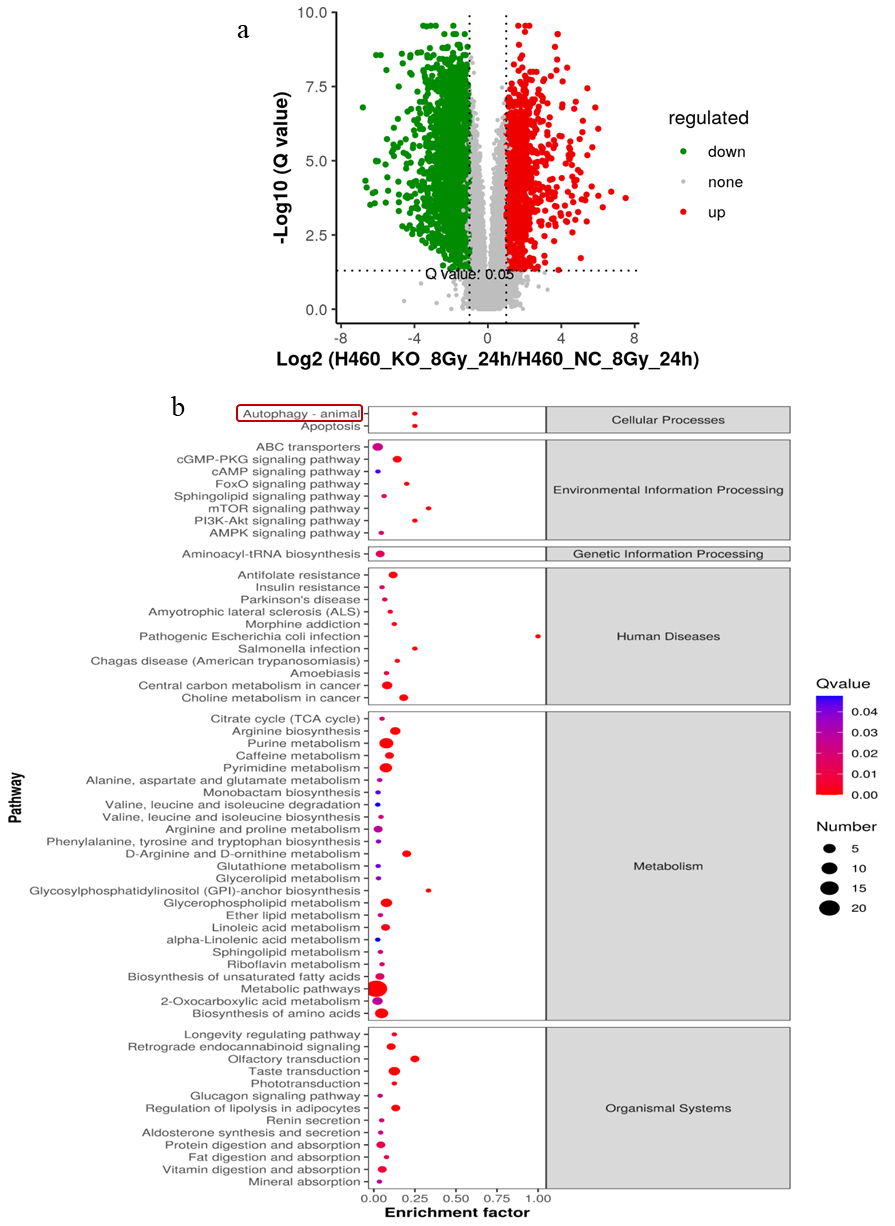


**Figure S13. Clustering and KEGG enrichment analysis of H460 NC/KO harvested 24h post 8 Gy IR based on the metabolomic source data.** **a** The volcano map shows the identification of differentially expressed genes in H460 NC/ KO cells 24h post 8Gy IR. **b** KEGG enrichment analysis of differential metabolites in H460 NC/KO cells 24h post 8Gy IR.


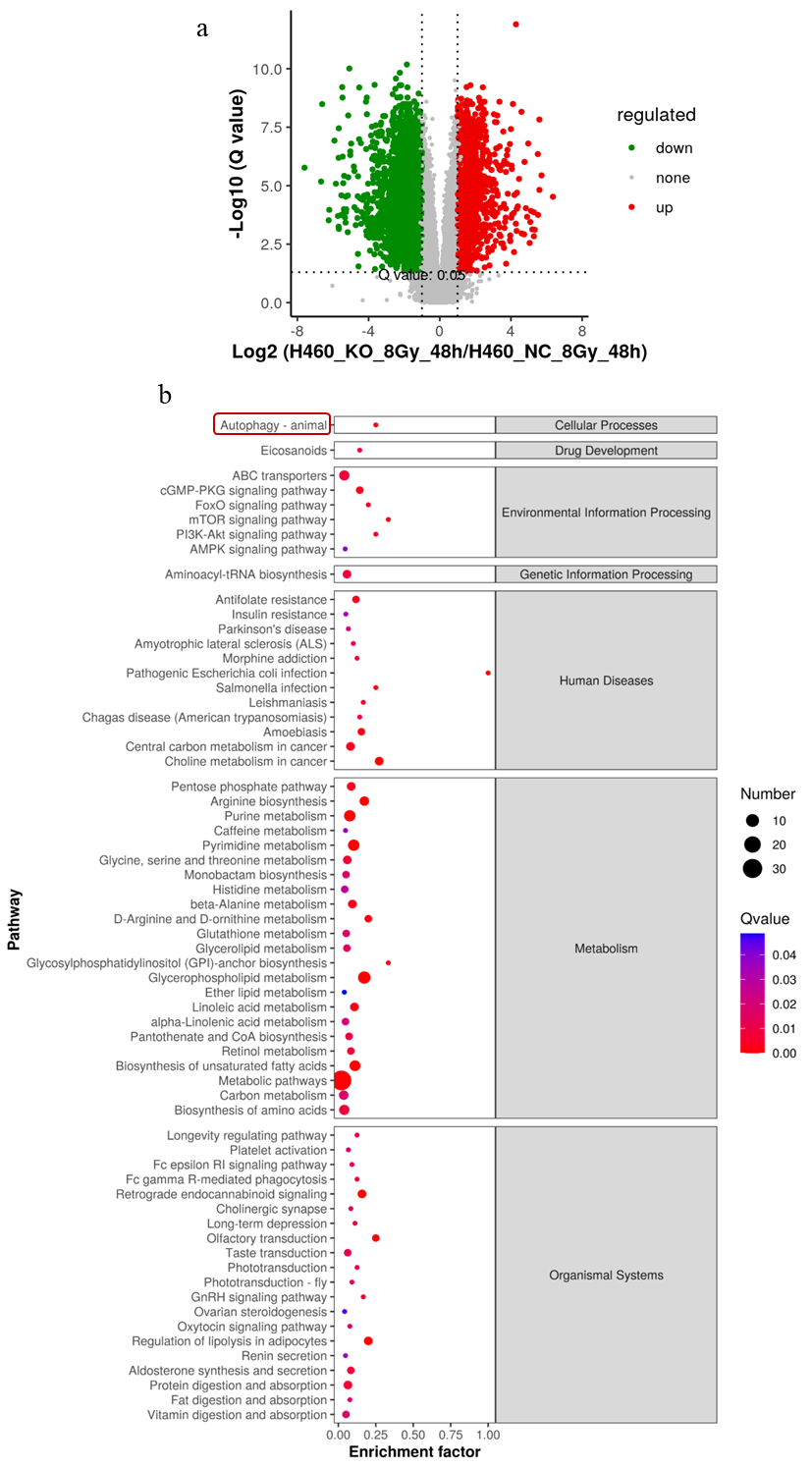


**Figure S14. Clustering and KEGG enrichment analysis of H460 NC/KO harvested 48h post 8Gy IR based on the metabolomic source data**. **a** The volcano map shows the identification of differentially expressed genes in H460 NC/ KO cells 48h post 8Gy IR. **b** KEGG enrichment analysis of differential metabolites in H460 NC/KO cells 48h post 8Gy IR.


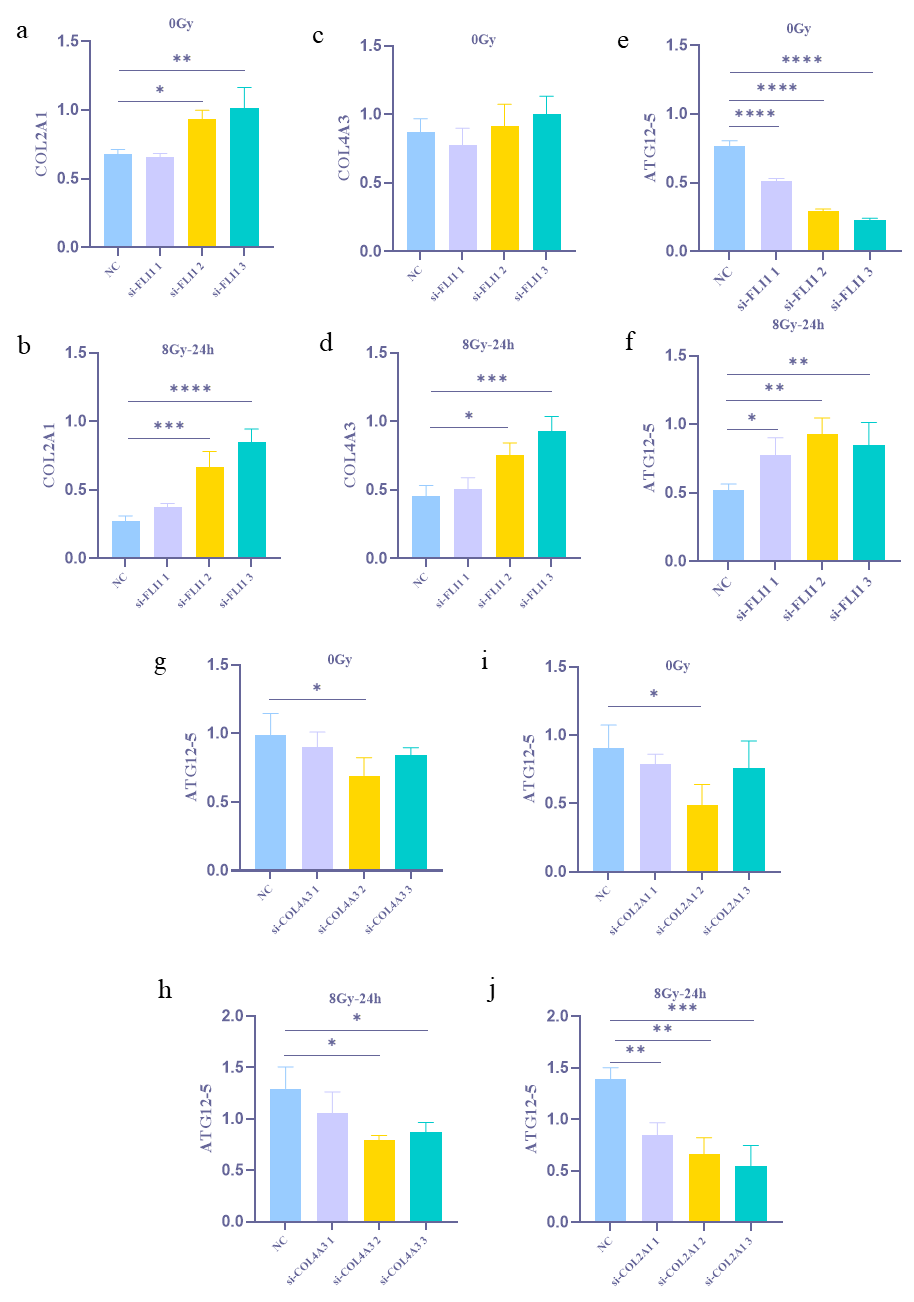


**Figure S15.** **Quantification and comparison of the protein levels of COLs and ATG12-5 in differently transfected H460 cells.** **a-f** The relative protein levels of COL2A1 and COL4A3 was quantified in H460 FLI1-NC/KD cells after sham/IR treatment. **g, h** The relative protein levels of ATG12-5 are shown using bar graphs in H460 COL4A3-NC/KD cells after sham/IR treatment. **i, j** The relative protein levels of ATG12-5 are shown using bar graphs in H460 COL2A1-NC/KD cells after sham/IR treatment.
